# Powdered and beaded sawdust materials modified iron (III) oxide-hydroxide for adsorption of lead (II) ion and reactive blue 4 dye

**DOI:** 10.1038/s41598-023-27789-9

**Published:** 2023-01-11

**Authors:** Pornsawai Praipipat, Pimploy Ngamsurach, Sichon Kosumphan, Jirasak Mokkarat

**Affiliations:** 1 Department of Environmental Science, Khon Kaen University, Khon Kaen, 40002 Thailand; 2 Environmental Applications of Recycled and Natural Materials (EARN) Laboratory, Khon Kaen University, Khon Kaen, 40002 Thailand

**Keywords:** Engineering, Materials science

## Abstract

The problems of lead and reactive blue 4 (RB4) dye contamination in wastewater are concerns because of their toxicities to aquatic life and water quality, so lead and RB4 dye removals are recommended to remove from wastewater before discharging. Sawdust powder (SP), sawdust powder doped iron (III) oxide-hydroxide (SPF), sawdust beads (SPB), and sawdust powder doped iron (III) oxide-hydroxide beads (SPFB) were synthesized and characterized with various techniques, and their lead or RB4 dye removal efficiencies were investigated by batch experiments, adsorption isotherms, kinetics, and desorption experiments. SPFB demonstrated higher specific surface area (11.020 m^2^ g^−1^) and smaller pore size (3.937 nm) than other materials. SP and SPF were irregular shapes with heterogeneous structures whereas SPB and SPFB had spherical shapes with coarse surfaces. Calcium (Ca) and oxygen (O) were found in all materials whereas iron (Fe) was only found in SPF and SPFB. O–H, C–H, C=C, and C–O were detected in all materials. Their lead removal efficiencies of all materials were higher than 82%, and RB4 dye removal efficiencies of SPB and SPFB were higher than 87%. Therefore, adding iron (III) oxide-hydroxide and changing material form helped to improve material efficiencies for lead or RB4 dye adsorption. SP and SPB corresponded to Langmuir model related to a physical adsorption process whereas SPF and SPFB corresponded to the Freundlich model correlated to a chemisorption process. All materials corresponded to a pseudo-second-order kinetic model relating to the chemical adsorption process. All materials could be reused more than 5 cycles with high lead removal of 63%, and SPB and SPFB also could be reused more than 5 cycles for high RB4 dye removal of 72%. Therefore, SPFB was a potential material to apply for lead or RB4 dye removal in industrial applications.

## Introduction

Water pollutions from heavy metals or dye contaminations create many problems with water quality, toxicity to aquatic life and the environment, a decrease of oxygen in water sources, an obstacle to sunlight for photosynthesis, and persistent, accumulation, and transport through the food chain. In addition, they also create many human health effects of dysfunctions in human systems such as the brain, blood, reproduction, digestive, and respiratory and cause cancers^1^. Especially, lead (Pb) is a toxic heavy metal with concern its toxicity with persistent and bioaccumulation. A reactive blue 4 (RB4) dye is popularly used in the textile industry because it offers long-lasting color in the fabric. However, if it is released into the environment without treatment, it affects aquatic life and the environment as mentioned above. The sources of releasing lead or RB4 dye are various industries of battery, electronic, paint, dye, plastic, and textile using them in their manufacturing processes^2,3^, so their wastewater might consist of lead or RB4 dye. As a result, wastewater with lead or dye contamination is required for a treatment to be below water quality standards for safety purposes.

Many methods of chemical precipitation, coagulation-flocculation, electrochemical, ion exchange, and reverse osmosis are used for eliminating heavy metals or dyes in wastewater; however, they have limitations of incompletely heavy metal removals, complicated operations with expensive costs including creating toxic sludges with requiring disposals^4^. As a result, many studies attempted to find an alternative method with an effective and environmentally friendly instead of them. An adsorption method is a good method to solve the above problems because this method offers high heavy metal and dye removals, suitable cost, simple operation, and creating low sludge volume^5^. In addition, various available choices of adsorbents of this method are a good option for a user with considering which adsorbent is good for removing a target pollutant by using the considering criteria of available adsorbent in that area, water quality after treatment, and budget. Several adsorbents are used for eliminating specific a target metal or dye ion in wastewater such as activated carbon, chitosan, zeolite, fruit peels, and wastes of agriculture, food, and industrial; however, this study will focus on various wastes as low-cost adsorbents used for improving water quality along with reducing waste volumes in terms of waste management. The elimination of heavy metals or dyes from wastewater from various wastes is demonstrated in Table 1. Among those adsorbents, sawdust is a good offer because it has good chemical properties of cellulose, hemicellulose, lignin, pectin, hydroxyl, and carboxyl groups for good lead or RB4 dye adsorption in wastewater. Furthermore, using sawdust can reduce a huge of sawmill factory waste and help to manage the problem of waste disposal along with improving water quality by using sawdust as an adsorbent material. Although sawdust has good chemical properties for removing lead or RB4 dye, the material improvement method needs to study for increasing lead or RB4 dye removal efficiencies in case of high-strength lead or RB4 dye concentration in industrial applications.

**Table 1:** The elimination of heavy metals or dyes from wastewater from various industrial wastes.

Adsorbent materials	Heavy metal/dye	Dose	Contact time	Temperature (°C)	pH	Concentration (mg L^−1^)	Removal efficiency (%)	References
Paddy husk (Biochar)	Lead (Pb^2+^)	0.1 g	24 h	25	6	10	99.00	^15^
Paddy husk (Biochar)	Copper (Cu^2+^)	0.1 g	24 h	25	6	10	99.00	^15^
Paddy husk (Biochar)	Zinc (Zn^2+^)	0.1 g	24 h	25	7.5	10	99.00	^15^
Sawdust (Biochar)	Lead (Pb^2+^)	0.1 g	24 h	25	6	10	99.00	^15^
Sawdust (Biochar)	Copper (Cu^2+^)	0.1 g	24 h	25	6	10	99.00	^15^
Sawdust (Biochar)	Zinc (Zn^2+^)	0.1 g	24 h	25	7.5	10	99.00	^15^
Pine wood sawdust modified with maleic acid	Cadmium (Cd^2+^)	0.3 g L^−1^	2 h	130	6	130	–	^16^
Bagasse	Lead (Pb^2+^)	1 g	2 h	–	5.5	100	74.40–80.00	^17^
Bagasse	Cadmium (Cd^2+^)	1 g	2 h	–	5.5	100	66.70–82.35	^17^
Eggshell	Lead (Pb^2+^)	1 g	2 h	–	5.5	100	99.55	^17^
Eggshell	Cadmium (Cd^2+^)	1 g	2 h	–	5.5	100	53–75	^17^
Lemon peel powder	Lead (Pb^2+^)	4 g	6 h	25	5	100	86.00	^18^
Lemon peel beads	Lead (Pb^2+^)	5 g	6 h	25	5	100	97.00	^18^
Melon peels	Lead (Pb^2+^)	1.5 g L^−1^	1 h	30	7	10	98.50	^19^
Melon peels	Copper (Cu^2+^)	1.5 g L^−1^	1 h	30	6	10	99.10	^19^
Melon peels	Cadmium (Cd^2+^)	1.5 g L^−1^	1 h	30	6	10	99.20	^19^
*Cordia trichotoma* sawdust	Crystal violet	0.8 g L^−1^	2 h	55	7.5	200	82.22	^20^
Potato peels	Cibacron blue P3R	2 g L^−1^	3 h	25	2.2	30	76.41	^21^
Potato peels modified with phosphoric acid	Cibacron blue P3R	2 g L^−1^	3 h	25	2.2	30	88.60	^21^
Potato peels calcined at 800 °C	Cibacron blue P3R	0.6 g L^−1^	3 h	25	2.2	30	94.00	^21^
Banana peels	Reactive black 5	0.3 g	24 h	25	3	300	–	^22^
Banana peels	Cong red	0.3 g	24 h	25	3	300	–	^22^
Fava bean peels	Methylene blue	5 g L^−1^	24 h	27	5.8	50	70.00–80.00	^23^
Clinoptilolite modified with Fe_3_O_4_	Basic violet 16	0.5 g L^−1^	45 min	–	7	25	99.00	^24^
Activated carbon modified with Fe_3_O_4_	Reactive blue 19	1 g L^−1^	45 min	–	3	100	93.22	^25^
Activated carbon modified with zinc oxide	Reactive blue 19	1.5 g L^−1^	45 min	–	3	100	97.36	^26^
Activated carbon modified with zinc oxide	Reactive black 5	1.5 g L^−1^	45 min	–	3	100	73.36	^26^
Walnut peel activated carbon modified with zinc oxide	Acid blue 113	0.5 g L^−1^	15 min	–	3	100	98.20	^27^
Walnut peel activated carbon modified with zinc oxide	Eosin Y	1 g L^−1^	30 min	–	3	100	95.11	^28^
Walnut peel activated carbon modified with zinc oxide	Erythrosine B	1 g L^−1^	30 min	–	3	100	98.31	^28^

For material modifications, many previous studies have been reported for using a variety of metal oxides of iron (II or III) oxide (Fe_4_O_3_ or Fe_2_O_3_), zinc oxide (ZnO), titanium dioxide (TiO_2_), aluminum oxide (Al_2_O_3_), tin (IV) oxide (SnO_2_), and magnesium oxide (MgO) to increase surface area and pore volume of adsorbents offering to increase lead or RB4 dye removal efficiency^6–8^. The modified materials of sugarcane bagasse with Fe_2_O_3_, sawdust with ZnO, and lemon peels with iron (III) oxide-hydroxide have been used for lead removal^8–10^, and the modifications of rice bran with SnO_2_/Fe_3_O_4_, bagasse with zinc oxide, and lemon peels beads with iron (III) oxide-hydroxide have been applied for removing RB4 dye^11–13^. Moreover, the stability of the material is another point for applying industrial application, so the changing of material form from a powder form to a bead form in many studies has also been reported with supporting the increase of heavy metal or dye removal efficiencies^6–8,12–14^. Therefore, this study attempts to synthesize sawdust materials modified with iron (III) oxide-hydroxide in powder and bead materials, compare their lead or RB4 dye removal efficiencies through batch experiments, and verify whether adding metal oxide or changing form helped to improve a material efficiency for lead or RB4 adsorption.

The study aimed to synthesize four types of adsorbent materials which were sawdust powder (SP), sawdust powder doped iron (III) oxide-hydroxide (SPF), sawdust beads (SPB), and sawdust powder doped iron (III) oxide-hydroxide beads (SPFB). Several characterized techniques of Brunauer–Emmett–Teller (BET), field emission scanning electron microscopy and focus ion beam (FESEM-FIB) with energy dispersive X-ray spectrometer (EDX), and Fourier transform infrared spectroscopy (FT-IR) were used to investigate their specific surface area, pore volume, pore size, surface morphologies, chemical compositions, and chemical functional groups. Lead or RB4 dye removal efficiencies of SP, SPF, SPB, and SPFB were examined by batch experiments with varying doses, contact time, temperature, pH, and concentration. Moreover, linear and nonlinear adsorption isotherms of Langmuir, Freundlich, Temkin, and Dubinin–Radushkevich models and kinetics of pseudo-first-kinetic, pseudo-second-kinetic, Elovich, and intraparticle diffusion models were used for investigating their lead or RB4 dye adsorption patterns and mechanisms. Finally, the desorption experiments were also investigated for confirming the reusability of sawdust materials for lead or RB4 dye adsorption.

## Result and discussion

### The physical characteristics of sawdust materials

**Figure 1:**
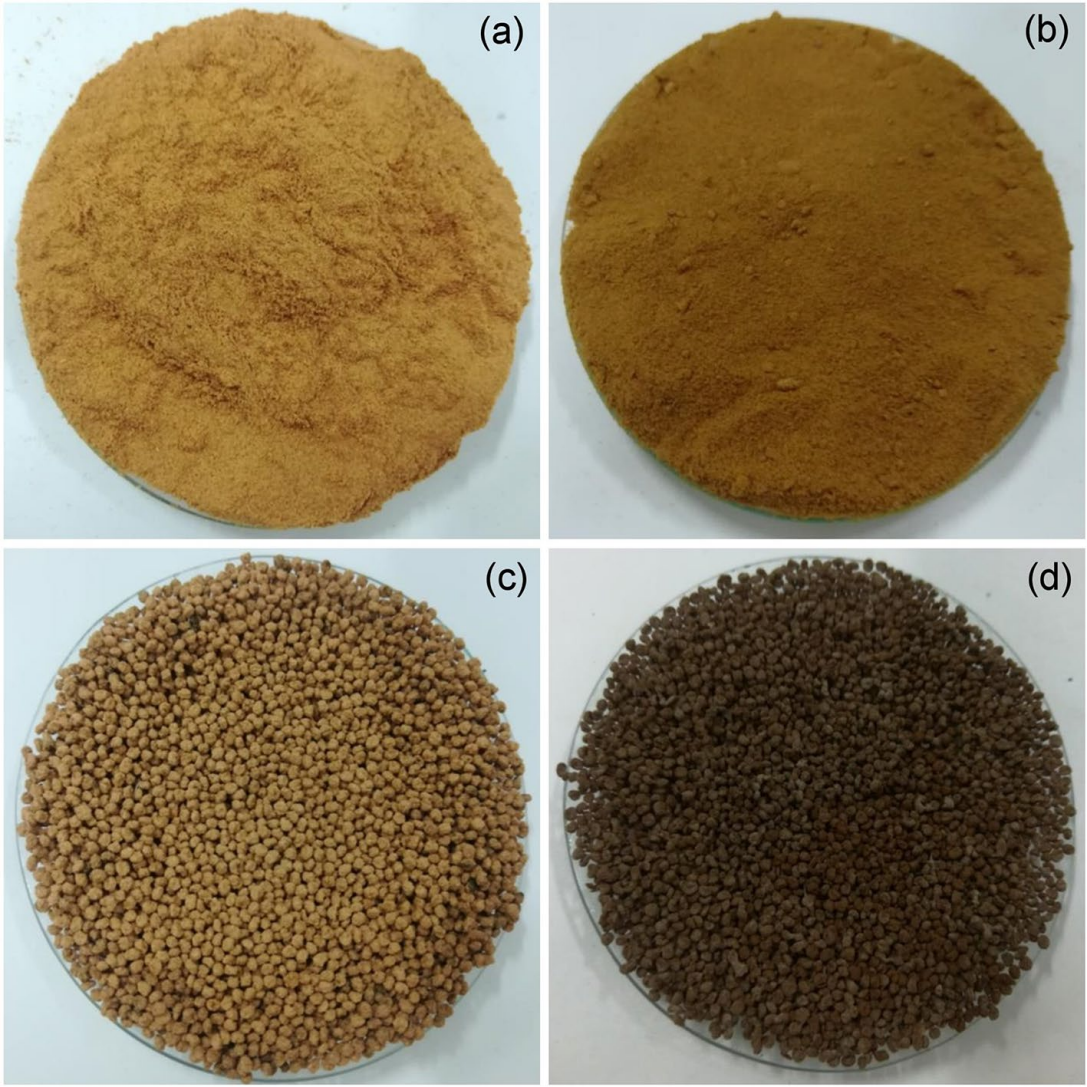
The physical characteristics of (**a**) sawdust powder (SP), (**b**) sawdust powder doped iron (III) oxide-hydroxide (SPF), (**c**) sawdust beads (SPB), and (**d**) sawdust powder doped iron (III) oxide-hydroxide beads (SPFB).

The physical characteristics of sawdust powder (SP), sawdust powder doped iron (III) oxide-hydroxide (SPF), sawdust beads (SPB), and sawdust powder doped iron (III) oxide-hydroxide beads (SPFB) are demonstrated in Fig. 1a–d. SP had light brown color powder whereas SPF had brown color powder shown in Fig. 1a,b. SPF had brown color beads while SPFB had dark brown color beads demonstrated in Fig. 1c,d.

### Characterizations of sawdust materials

#### BET analysis

The specific surface area, pore volume, and pore diameter size of sawdust powder (SP), sawdust powder doped iron (III) oxide-hydroxide (SPF), sawdust beads (SPB), and sawdust powder doped iron (III) oxide-hydroxide beads (SPFB) determined by the Brunauer–Emmet and Teller technique (BET) with N_2_ adsorption–desorption isotherm at 77.3 K and degas temperature of 80 °C for 6 h, and the results of specific surface area and pore volume by Brunauer–Emmett–Teller (BET) and pore size by Barrett–Joyner–Halenda (BJH) method are reported in Table 2.

**Table 2:** The specific surface area, pore volumes, and pore size of sawdust materials with comparing other studies.

Adsorbent materials	Surface area (m^2^ g^−1^)	Pore volume (cm^3^ g^−1^)	Pore diameter size (nm)	References
Sawdust	0.697	–	11.399	^29^
Sawdust	1.090	–	–	^30^
Palm date sawdust	1.360	–	0.500	^31^
Pine sawdust biochar	13.080	0.010	1.320	^32^
Sawdust modified by sodium hydroxide and triethanolamine	0.969	–	24.485	^29^
Wheat bran sawdust modified by Fe_3_O_4_	74.250	0.119	6.422	^33^
Unmodified sawdust	–	0.004	–	^34^
Unmodified sawdust mixed CoFe_2_O_4_	18.083	0.098	26.603	^34^
Unmodified sawdust mixed NiFe_2_O_4_	19.649	0.085	17.202	^34^
Modified sawdust	0.230	0.006	103.790	^34^
Modified sawdust mixed CoFe_2_O_4_	18.077	0.083	18.363	^34^
Modified sawdust mixed NiFe_2_O_4_	91.736	0.250	10.901	^34^
SP	0.328	0.075	4.256	This study
SPF	1.551	0.356	3.940	This study
SPB	0.960	0.250	4.068	This study
SPFB	11.020	2.532	3.937	This study

For the specific surface area of SP, SPF, SPB, and SPFB, they were 0.328, 1.551, 1.960, and 11.020 m^2^ g^−1^, respectively which SPFB demonstrated the highest specific surface area than other materials. In addition, their pore volumes were 0.075, 0.356, 0.250, and 2.532 cm^3^ g^−1^, respectively, and their pore diameter sizes were 4.256, 3.940, 4.068, and 3.937 nm, respectively. As a result, the addition of iron (III) oxide-hydroxide into sawdust materials (SPF and SPFB) increased the specific surface area and pore volume while the pore diameter size was decreased. In addition, all three parameters increased from changing material form from SP to SPB. Since their pore size was in a range of 2–50 nm, they were classified as mesoporous by the classification of International Union of Pure and Applied Chemistry (IUPAC)^35^.

For the BET comparison, SP had a lower specific surface area than all studies reported in Table 2 whereas SPF, SPB, and SPFB had higher values than the studies of Chen et al. and Houshangi et al.^29,34^. The studies of Chen et al. and Houshangi et al. had been reported that the modifications of sawdust materials by sodium hydroxide (NaOH) or triethanolamine (C_6_H_15_NO_3_) or iron dioxide (Fe_2_O_4_) helped to increase specific surface area similar to this study^29,34^.

#### FESEM-FIB analysis

**Figure 2:**
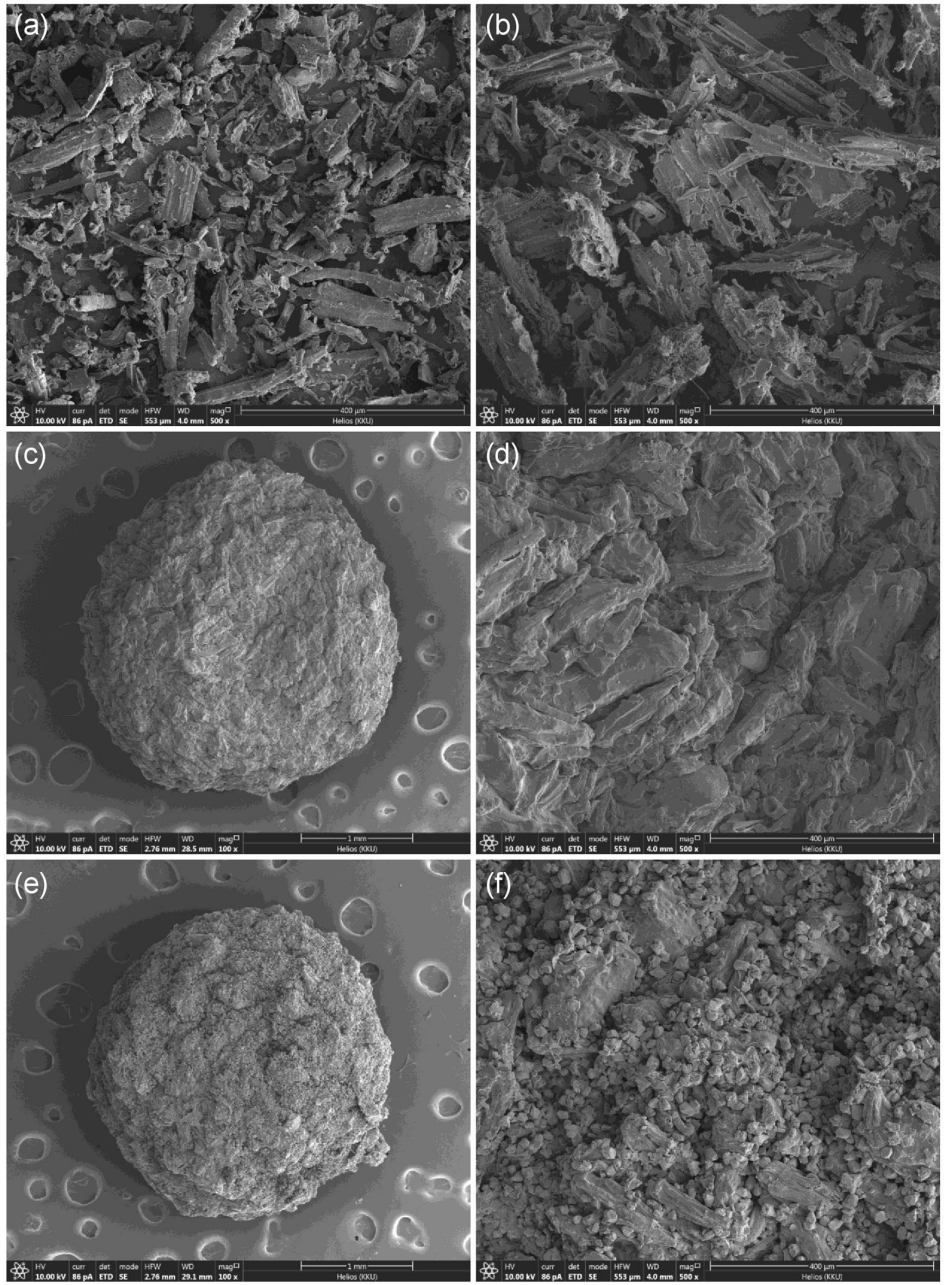
The surface morphologies of (**a**) sawdust powder (SP), (**b**) sawdust powder doped iron (III) oxide-hydroxide (SPF), (**c**,**d**) sawdust beads (SPB), and (**e**,**f**) sawdust powder doped iron (III) oxide-hydroxide beads (SPFB).

The surface morphologies of sawdust powder (SP), sawdust powder doped iron (III) oxide-hydroxide (SPF), sawdust beads (SPB), and sawdust powder doped iron (III) oxide-hydroxide beads (SPFB) by FESEM-FIB analysis at 500 × magnification with 400 µm for a surface and at 100 × magnification with 1 mm for a bead illustrated in Fig. 2a–f. SP and SPF were irregular shapes with heterogeneous fiber structures demonstrated in Fig. 2a,b similar reported of sawdust morphologies by other studies^36,37^. For SPB, it had a spherical shape with a coarse surface at 100 × magnification with 1 mm shown in Fig. 2c, and its surface was a rough surface when zoomed at 500 × magnification with 400 µm demonstrated in Fig. 2d. Finally, SPFB had a spherical shape with a coarse surface at 100 × magnification of 1 mm shown in Fig. 2e, and its surface was an irregular shape with the heterogenous surface when zoomed at 500 × magnification with 400 µm illustrated in Fig. 2f.

#### EDX analysis

**Figure 3:**
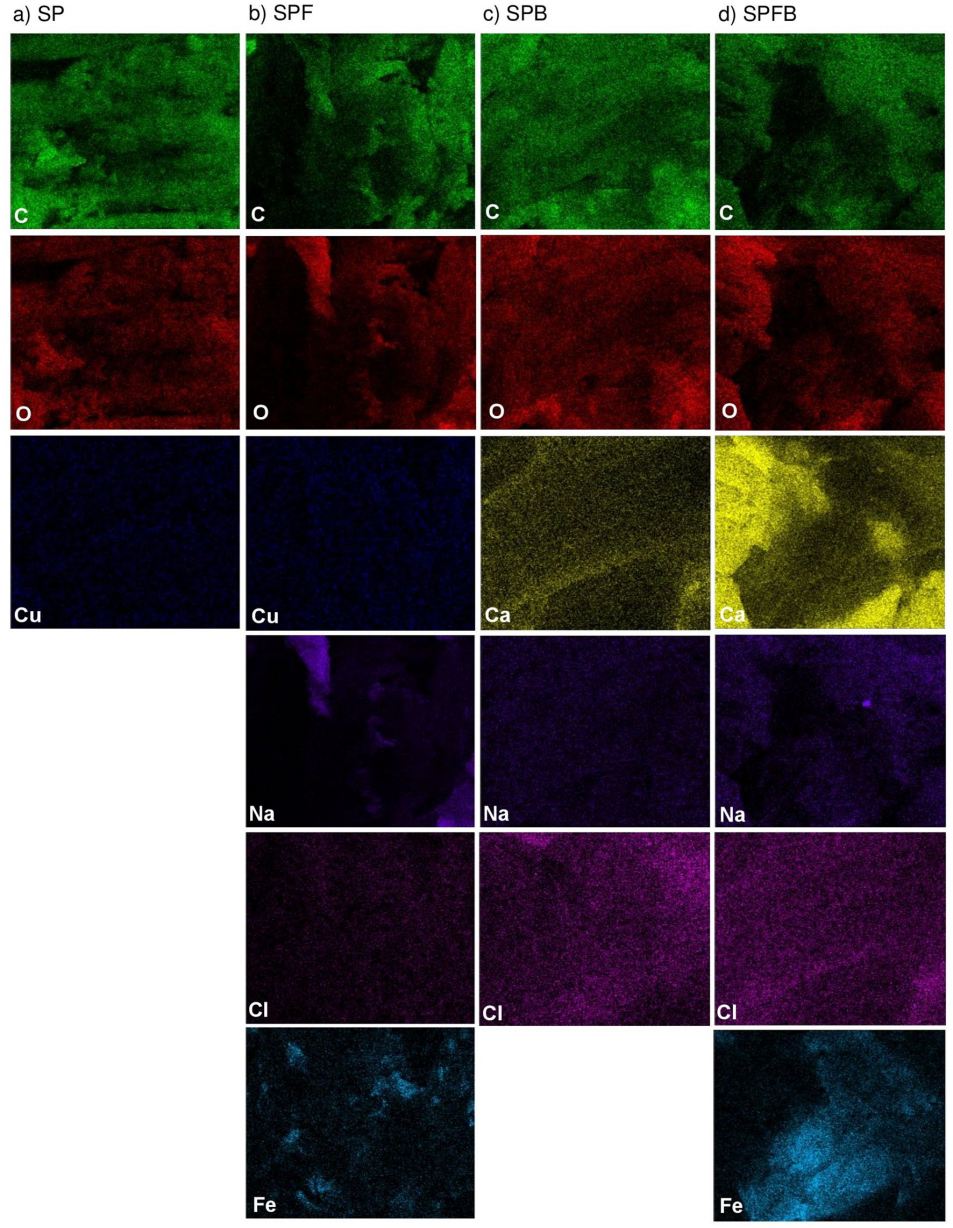
The elemental mapping of (**a**) sawdust powder (SP), (**b**) sawdust powder doped iron (III) oxide-hydroxide (SPF), (**c**) sawdust beads (SPB), and (**d**) sawdust powder doped iron (III) oxide-hydroxide beads (SPFB) on the surface.

The chemical compositions of sawdust powder (SP), sawdust powder doped iron (III) oxide-hydroxide (SPF), sawdust beads (SPB), and sawdust powder doped iron (III) oxide-hydroxide beads (SPFB) were analyzed by using EDX analysis represented in Table 3, and the elemental mapping of SP, SPF, SPB, and SPFB demonstrated in Fig. 3a–d which showed the dispersions of chemical elements of each material on the surface. Two main chemical components of carbon (C) and oxygen (O) were found in all materials while copper (Cu) was only found in powder materials of SP and SPF. For calcium (Ca), it was detected in SPB and SPFB. For sodium (Na) and chloride (Cl), they were observed in all materials except SP. In addition, iron (Fe) was found in the materials with the addition of iron (III) oxide-hydroxide which were SPF and SPFB to confirm the successful adding Fe into SP and SPB. For SP and SPF, the mass percentages by weight of C and O were decreased when iron (III) oxide-hydroxide was added to SP whereas Cu was increased. Moreover, Na, Cl, and Fe were detected in SPF which might be from using chemicals in a process of adding iron (III) oxide-hydroxide by ferric chloride hexahydrate (FeCl_3_^.^6H_2_O) and sodium hydroxide (NaOH) for synthesizing SPF. For SP and SPB, the mass percentage by weight of C was deceased while O was increased after changing the material to a bead form. In addition, the mass percentages by weight of Ca, Na, and Cl were also detected in SPB by using sodium alginate (NaC_6_H_7_O_6_) and calcium chloride (CaCl_2_) in a bead formation. For SPF and SPFB, the mass percentages by weight of C, O, and Na were decreased. While the mass percentages by weight of Ca, Cl, and Fe were increased when SPF was changed to a bead form. The increases of Ca and Cl might be from using chemicals of CaCl_2_ in a process of a bead formation similar to SPB. Therefore, the addition of iron (III) oxide-hydroxide and the changing material form affected the increases of Ca, Na, Cl, and Fe contents in sawdust materials.

**Table 3:** The chemical compositions of sawdust powder (SP), sawdust powder doped iron (III) oxide-hydroxide (SPF), sawdust beads (SPB), and sawdust powder doped iron (III) oxide-hydroxide beads (SPFB) in the percentages by weight.

Chemical composition (%wt)	Sawdust materials			
	SP	SPF	SPB	SPFB
C	59.6	48.2	51.7	32.6
O	39.0	38.1	40.5	31.0
Cu	1.4	1.5	–	–
Ca	–	–	5.1	23.2
Na	–	8.4	0.5	0.7
Cl	–	0.2	2.2	1.3
Fe	–	3.6	–	11.3

#### FT-IR analysis

**Figure 4:**
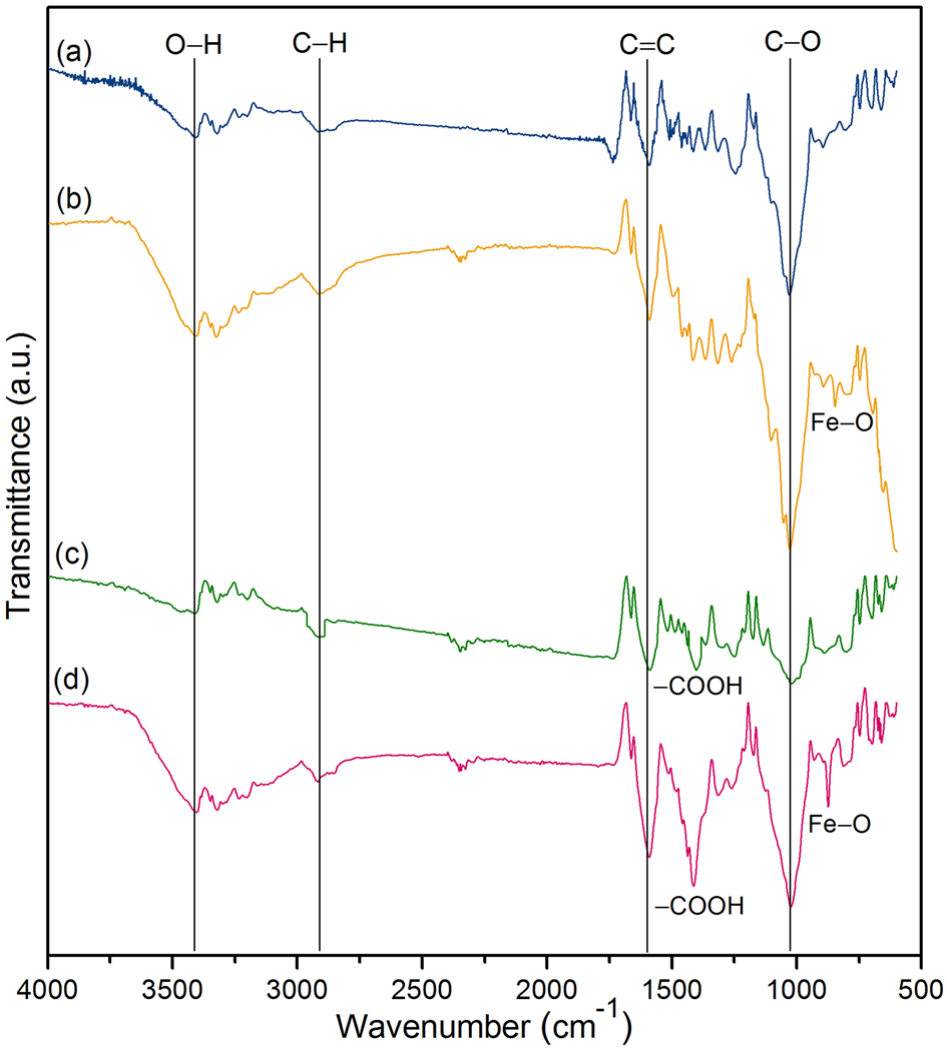
FT-IR spectra of (**a**) sawdust powder (SP), (**b**) sawdust powder doped iron (III) oxide-hydroxide (SPF), (**c**) sawdust beads (SPB), and (**d**) sawdust powder doped iron (III) oxide-hydroxide beads (SPFB).

The chemical functional groups of sawdust powder (SP), sawdust powder doped iron (III) oxide-hydroxide (SPF), sawdust beads (SPB), and sawdust powder doped iron (III) oxide-hydroxide beads (SPFB) were examined by FT-IR analysis, and their FT-IR spectra are demonstrated in Fig. 4a–d. Four main function groups of O–H, C–H, C=C, and C–O were detected in all materials. In addition, the carboxyl group of sodium alginate (–COOH) was found in bead materials (SPB and SPFB)^38^, and Fe–O was observed in materials with adding iron (III) oxide-hydroxide (SPF and SPFB)^39^. O–H represented the stretching of hydroxyl, alcohol, and phenolic groups of cellulose fiber, lignin, and pectin, and C–H demonstrated the stretching of a methyl group (–CH_2_) in cellulose and hemicellulose^40^. C=C referred to the stretching of aromatic rings corresponding to the lignin, and C–O was the stretching of alcohol and carboxylic acid of lignin and hemicellulose^41^. For SP, it detected the stretching of O–H at 3322.83 cm^−1^, stretching of C–H at 2918.28 cm^−1^, stretching of C=C at 1666.59 cm^−1^, and stretching of C–O at 1245.34 and 1030.31 cm^−1^ illustrated in Fig. 4a. For SPF, it observed the stretching of O–H at 3325.99 cm^−1^, stretching of C–H at 2916.52 cm^−1^, C=C at 1664.48 cm^−1^, stretching of C–O at 1262.29 and 1028.88 cm^−1^, and Fe–O at 847.36 cm^−1^ shown in Fig. 4b. For SPB, it identified the stretching of O–H at 3323.08 cm^−1^, C–H at 2910.48 cm^−1^, C=C at 1664.90 cm^−1^, stretching of C–O at 1249.35 and 1021.42 cm^−1^, and –COOH at 1419.56 cm^−1^ shown in Fig. 4c. For SPFB, it found the stretching of O–H at 3323.68 cm^−1^, stretching of C–H at 2919.56 cm^−1^, C=C at 1664.33 cm^−1^, stretching of C–O at 1262.71 and 1024.87 cm^−1^, –COOH at 1421.49 cm^−1^, and Fe–O at 813.34 cm^−1^ shown in Fig. 4d.

### Batch adsorption experiments for lead and RB4 dye removals

#### The effect of dose

**Figure 5:**
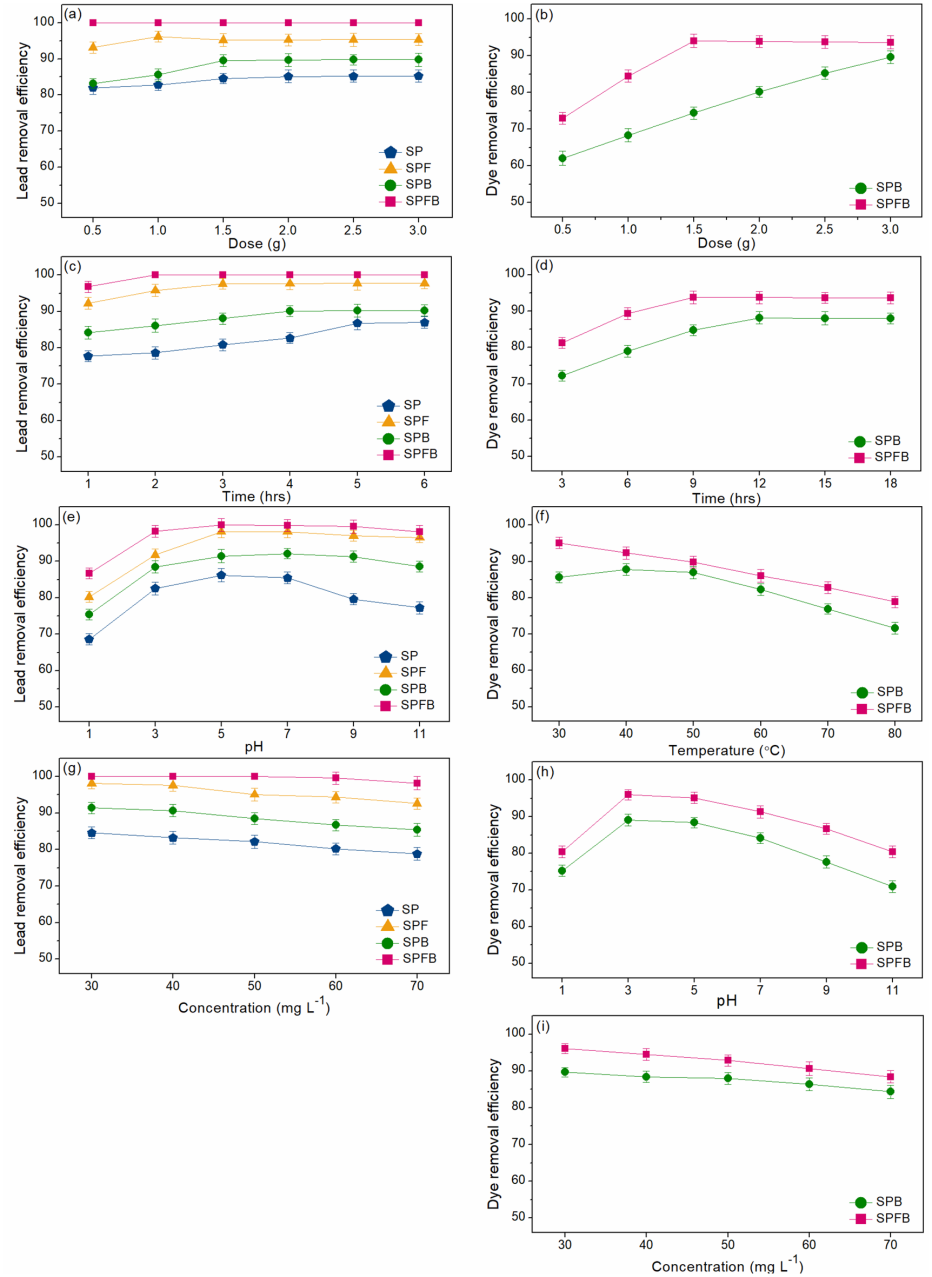
Batch experiments for lead and RB4 dye removals on the effects of (**a**,**b**) dose, (**c**,**d**) contact time, (**f**) temperature, (**e**,**h**) pH, and (**g**,**i**) concentration of sawdust powder (SP), sawdust powder doped iron (III) oxide-hydroxide (SPF), sawdust beads (SPB), and sawdust powder doped iron (III) oxide-hydroxide beads (SPFB).

For lead removal, five different doses from 0.5 to 3 g were used for investigating the dose effect of lead adsorption by sawdust powder (SP), sawdust powder doped iron (III) oxide-hydroxide (SPF), sawdust beads (SPB), and sawdust powder doped iron (III) oxide-hydroxide beads (SPFB), and the results are demonstrated in Fig. 5a. The control condition was the lead concentration of 50 mg L^−1^, a sample volume of 200 mL, a contact time of 6 h, pH 5, a temperature of 25 °C, and a shaking speed of 200 rpm. Lead removal efficiencies of all materials were increased with the increase of material dose which might be from the increase of active sites of materials^7^. Their highest lead removal efficiencies were 85.12%, 96.11%, 89.57%, and 100% at 2 g, 1 g, 1.5 g, and 0.5 g for SP, SPF, SPB, and SPFB, respectively. Therefore, they were optimum doses of sawdust materials that were used for studying the contact time effect.

For RB4 dye removal, six different doses from 0.5 to 3 g were used for investigating the dose effect of RB4 dye adsorption by sawdust beads (SPB) and sawdust powder doped iron (III) oxide-hydroxide beads (SPFB), and the results are demonstrated in Fig. 5b. The control condition was the RB4 dye concentration of 50 mg L^−1^, a sample volume of 200 mL, a contact time of 12 h, pH 7, a temperature of 60 °C, and a shaking speed of 150 rpm. RB4 dye removal efficiencies of all materials were increased with the increase of material dose which might be from the increase of active sites of materials^12^. The highest RB4 dye removal efficiency of SPB was found at 3 g with 89.65% while the highest RB4 dye removal efficiency of SPFB was found at 1.5 g with 94.10%. Therefore, they were the optimum dosages of sawdust materials that were used for studying the contact time effect.

#### The effect of contact time

For lead removal, the different contact times from 1 to 6 h were used for studying the contact time effect on lead adsorptions by sawdust powder (SP), sawdust powder doped iron (III) oxide-hydroxide (SPF), sawdust beads (SPB), and sawdust powder doped iron (III) oxide-hydroxide beads (SPFB), and the results are demonstrated in Fig. 5c. The control condition was the lead concentration of 50 mg L^−1^, a sample volume of 200 mL, pH 5, a temperature of 25 °C, a shaking speed of 200 rpm, and the optimum dose of 2 g (SP) or 1 g (SPF) or 1.5 g (SPB) or 0.5 g (SPFB). Lead removal efficiencies of all materials were increased with the increase of contact time similar to the dose effect. Their highest lead removal efficiencies were 86.74%, 97.58%, 90.12%, and 100% at 5 h, 3 h, 4 h, and 2 h for SP, SPF, SPB, and SPFB, respectively. Therefore, they were the optimum contact time of sawdust materials that were used for studying the pH effect.

For RB4 dye removal, the different contact times from 3 to 18 h were used for studying the contact time effect on RB4 dye adsorptions by sawdust beads (SPB) and sawdust powder doped iron (III) oxide-hydroxide beads (SPFB), and the results are demonstrated in Fig. 5d. The control condition was the RB4 dye concentration of 50 mg L^−1^, a sample volume of 200 mL, pH 7, a temperature of 60 °C, a shaking speed of 150 rpm, and the optimum dose 3 g (SPB) or 1.5 g (SPFB). RB4 dye removal efficiencies of all materials were increased with the increase of contact time similar to the dose effect. Their highest RB4 dye removal efficiencies were found at 12 h with 88.15% for SPB and 9 h with 93.76% for SPFB. Therefore, they were the optimum contact times of sawdust materials that were used for studying the pH effect.

#### The effect of temperature effect

Only dye removal investigated the effect of temperature whether the changing temperature affects RB4 dye removal by sawdust beads (SPB) and sawdust powder doped iron (III) oxide-hydroxide beads (SPFB). The different temperatures from 40 to 80 °C were used for studying the temperature effect on RB4 dye adsorptions by sawdust materials, and the results are demonstrated in Fig. 5f. The control condition was the RB4 dye concentration of 50 mg L^−1^, a sample volume of 200 mL, pH 7, a shaking speed of 150 rpm, and the optimum dose of 3 g (SPB) or 1.5 g (SPFB) and contact time of 12 h (SPB) or 9 h (SPFB). RB4 dye removal efficiencies of all materials were decreased with the increase of temperature, and the highest RB4 dye removal efficiencies were at a temperature of 40 °C with 87.78% for SPB and 30 °C with 95.12% for SPFB. Therefore, they were the optimum temperatures of sawdust materials that were used for studying the pH effect.

#### The effect of pH

For lead removal, the effect of pH was studied by varying pH values of 1, 3, 5, 7, 9, and 11 represented the acid, neutral, and base conditions on lead adsorptions by sawdust powder (SP), sawdust powder doped iron (III) oxide-hydroxide (SPF), sawdust beads (SPB), and sawdust powder doped iron (III) oxide-hydroxide beads (SPFB), and the results are demonstrated in Fig. 5e. The control condition was the lead concentration of 50 mg L^−1^, a sample volume of 200 mL, a temperature of 25 °C, a shaking speed of 200 rpm, and the optimum dose of 2 g (SP) or 1 g (SPF) or 1.5 g (SPB) or 0.5 g (SPFB) and contact time of 5 h (SP) or 3 h (SPF) or 4 h (SPB) or 2 h (SPFB). Lead removal efficiencies of all materials were increased with the increase of pH values from 1 to 7, then they were decreased. Their highest lead removal efficiencies of all materials were found at pH 5 with lead removal at 86.21%, 98.15%, 91.45%, and 100% for SP, SPF, SPB, and SPFB, respectively which corresponded to other previous studies reported the highest lead removal efficiency at pH > 4^7,8,18^. Therefore, pH 5 was the optimum pH of sawdust materials that were used for studying the concentration effect.

For RB4 dye removal, the effect of pH was studied by varying pH values of 1, 3, 5, 7, 9, and 11 represented the acid, neutral, and base conditions on RB4 dye adsorptions by sawdust beads (SPB) and sawdust powder doped iron (III) oxide-hydroxide beads (SPFB), and the results are demonstrated in Fig. 5h. The control condition was the RB4 dye concentration of 50 mg L^−1^, a sample volume of 200 mL, a shaking speed of 150 rpm, and the optimum dose 3 g (SPB) or 1.5 g (SPFB), contact time of 12 h (SPB) or 9 h (SPFB), and temperature of 40 °C (SPB) or 30 °C (SPFB). RB4 dye removal efficiencies of all materials were increased with the increase of pH values from 1 to 3, then they were decreased. Their highest RB4 dye removal efficiencies of all materials were found at pH 3 with RB4 dye removal at 89.12% and 95.96% for SPB and SPFB which corresponded to other previous studies reported the highest RB4 dye removal efficiency found at acidic conditions^6,14^. Therefore, pH 3 was the optimum pH of sawdust materials that were used for studying the concentration effect.

#### The effect of concentration

For lead removal, lead concentrations of 30–70 mg L^−1^ were used to investigate the concentration effect on lead adsorptions by sawdust powder (SP), sawdust powder doped iron (III) oxide-hydroxide (SPF), sawdust beads (SPB), and sawdust powder doped iron (III) oxide-hydroxide beads (SPFB), and the results are demonstrated in Fig. 5g. The control condition was a sample volume of 200 mL, a temperature of 25 °C, a shaking speed of 200 rpm, and the optimum dose of 2 g (SP) or 1 g (SPF) or 1.5 g (SPB) or 0.5 g (SPFB) and contact time of 5 h (SP) or 3 h (SPF) or 4 h (SPB) or 2 h (SPFB), and pH of 5. Lead removal efficiencies of all materials were decreased with the increase of concentration because of the decreasing available active sites for adsorbing lead ions^7^. Lead removal efficiencies from 30 to 70 mg L^−1^ of SP, SPF, SPB, and SPFB were 78.82–84.56%, 92.57–98.12%, 85.41–91.39%, and 97.15–100%, respectively. For the lead concentration of 50 mg L^−1^, lead removal efficiencies of SP, SPF, SPB, and SPFB were 82.14%, 95.06%, 88.45%, and 100%, respectively, and SPFB demonstrated the highest lead removal efficiency of other materials.

For RB4 dye removal, RB4 dye concentrations of 30–70 mg L^−1^ were used to investigate the concentration effect on RB4 dye adsorptions by sawdust beads (SPB) and sawdust powder doped iron (III) oxide-hydroxide beads (SPFB), and the results are demonstrated in Fig. 5i. The control condition was a sample volume of 150 mL, and the optimum dose of 3 g (SPB) or 1.5 g (SPFB), contact time of 12 h (SPB) or 9 h (SPFB), temperature of 40 °C (SPB) or 30 °C (SPFB), and pH of 3. RB4 dye removal efficiencies of all materials were decreased with the increase of concentration because RB4 dye ions were more than the available active sites of sawdust materials similar to the report by other studies^12,14^. RB4 dye removal efficiencies from 30 to 70 mg L^−1^ of SPB and SPFB were 84.35–89.65% and 88.43–96.12%. For the RB4 dye concentration of 50 mg L^−1^, RB4 dye removal efficiencies of SPB and SPFB were 87.96% and 92.84%, and SPFB demonstrated the highest RB4 dye removal efficiency of other materials.

In conclusion of lead removal, 2 g, 5 h, pH 5, 50 mg L^−1^, 1 g, 3 h, pH 5, 50 mg L^−1^, 1.5 g, 4 h, pH 5, 50 mg L^−1^, and 0.5 g, 2 h, pH 5, 50 mg L^−1^ were the optimum conditions in dose, contact time, pH, and concentration of SP, SPF, SPB, and SPFB, respectively, and they could be arranged in order from high to low of SPFB > SPF > SPB > SP. As a result, both changing material form and adding iron (III) oxide-hydroxide helped to improve material efficiency for lead adsorption.

In conclusion of RB4 dye removal, 3 g, 12 h, 40 °C, pH 3, 50 mg L^−1^ and 1.5 g, 9 h, 30 °C, pH 3, 50 mg L^−1^ were the optimum conditions in dose, contact time, temperature, pH, and concentration of SPB and SPFB, respectively. As a result, the changing material form and adding iron (III) oxide-hydroxide helped to improve material efficiency for RB4 dye adsorption than only changing material form.

Therefore, sawdust materials could be removed both lead and RB4 dye in aqueous solutions, and SPFB demonstrated the highest removal efficiency on both pollutants. Finally, SPFB was recommended to be applied for lead or RB4 dye removal in the wastewater treatment system in the future.

### Adsorption isotherms for lead and RB4 dye adsorptions

**Figure 6:**
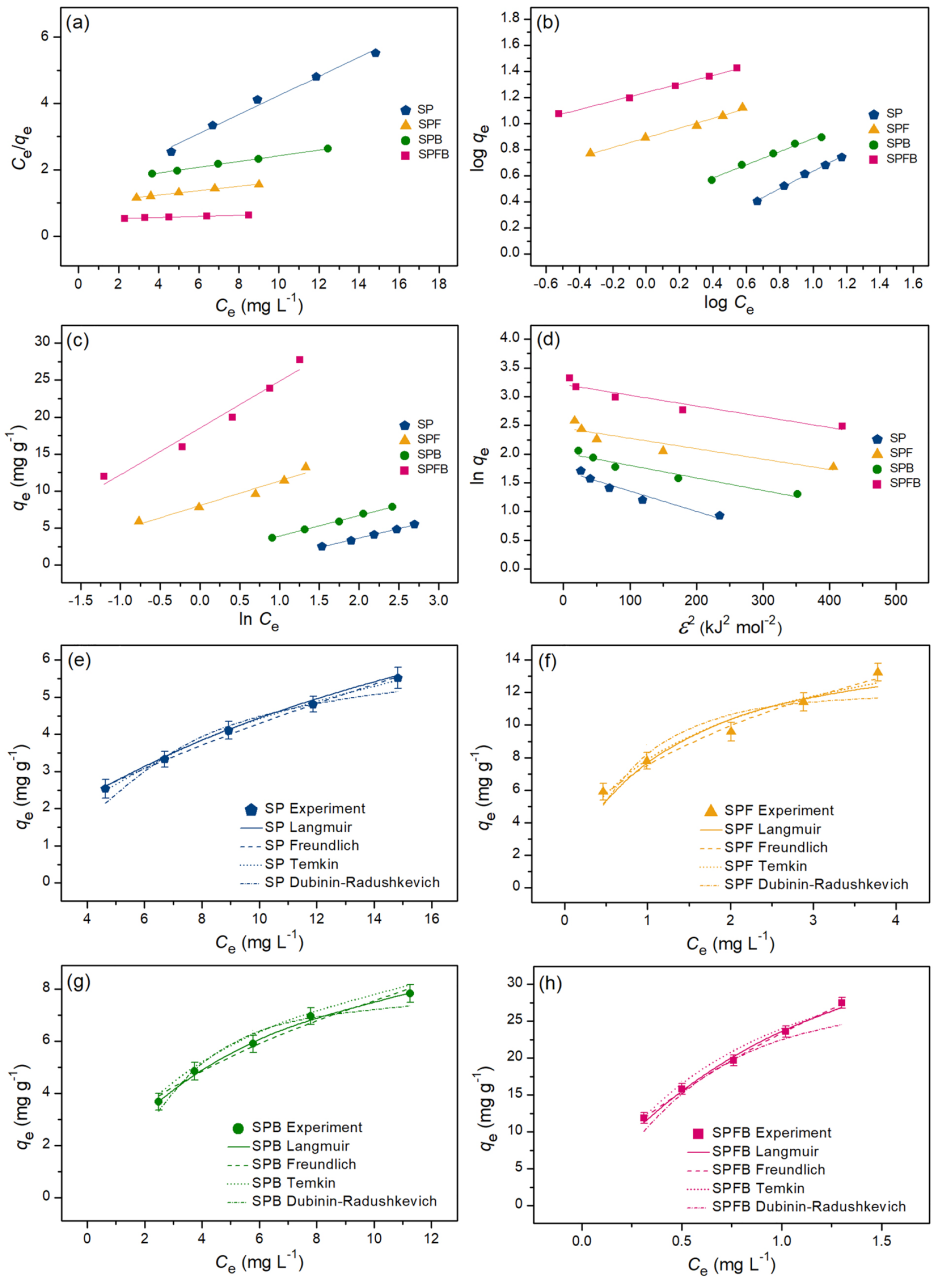
Graphs of (**a**) linear Langmuir, (**b**) linear Freundlich, (**c**) linear Temkin, (**d**) linear Dubinin–Radushkevich, and (**e**–**h**) nonlinear adsorption isotherms of sawdust powder (SP), sawdust powder doped iron (III) oxide-hydroxide (SPF), sawdust beads (SPB), and sawdust powder doped iron (III) oxide-hydroxide beads (SPFB) for lead adsorptions.

**Figure 7:**
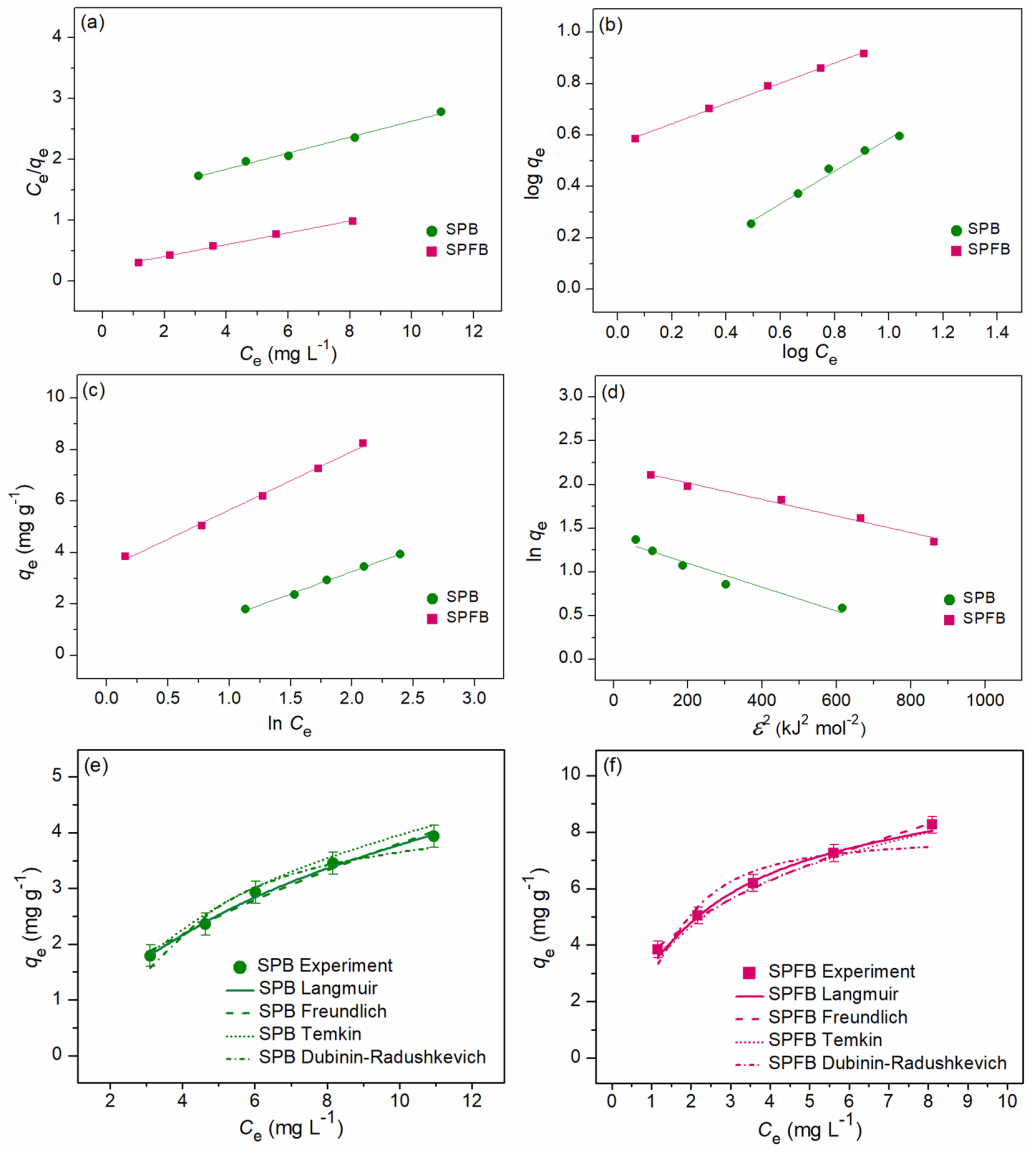
Graphs of (**a**) linear Langmuir, (**b**) linear Freundlich, (**c**) linear Temkin, (**d**) linear Dubinin–Radushkevich, and (**e**,**f**) nonlinear adsorption isotherms of sawdust beads (SPB), and sawdust powder doped iron (III) oxide-hydroxide beads (SPFB) for RB4 dye adsorptions.

The adsorption patterns of sawdust powder (SP), sawdust powder doped iron (III) oxide-hydroxide (SPF), sawdust beads (SPB), and sawdust powder doped iron (III) oxide-hydroxide beads (SPFB) for lead adsorption and sawdust beads (SPB) and sawdust powder doped iron (III) oxide-hydroxide beads (SPFB) for RB4 dye adsorption were investigated through various adsorption isotherms of Langmuir, Freundlich, Temkin, and Dubinin–Radushkevich models in both linear and nonlinear models. For linear models, Langmuir, Freundlich, Temkin, and Dubinin–Radushkevich isotherms were plotted by *C*_e_/*q*_e_ versus *C*_e_, log *q*_e_ versus log *C*_e_, *q*_e_ versus ln *C*_e_, and ln *q*_e_ versus *ε*^2^, respectively. For nonlinear models, all isotherms were plotted by *C*_e_ versus *q*_e._ The plotting graphs of lead and RB4 dye adsorption demonstrated in Figs. 6a–h and 7a–f, respectively, and their isotherm parameters were illustrated in Tables 4 and 5, respectively. Generally, the best-fit isotherm model for explaining the adsorption pattern of material is chosen from the high regression value (*R*^2^) which is close to 1^12^.

**Table 4:** The comparison of linear and nonlinear isotherm parameters for lead adsorptions on sawdust powder (SP), sawdust powder doped iron (III) oxide-hydroxide (SPF), sawdust beads (SPB), and sawdust powder doped iron (III) oxide-hydroxide beads (SPFB).

Regression method	Isotherm model	Parameter	SP	SPF	SPB	SPFB
Linear	Langmuir	*q*_m_ (mg g^−1^)	11.737	15.924	12.771	47.170
		*K*_L_ (L mg^−1^)	0.059	0.992	0.155	1.014
		*R*^2^	0.998	0.970	0.998	0.981
	Freundlich	1*/n*	0.666	0.372	0.503	0.128
		*K*_F_ (mg g^−1^) (L mg^−1^)^1/n^	0.930	7.773	2.411	11.495
		*R*^2^	0.996	0.990	0.986	0.994
	Temkin	*b*_T_ (J mol^−1^)	970.639	745.622	750.520	1040.471
		*A*_T_ (L g^−1^)	0.566	11.297	1.414	24.249
		*R*^2^	0.996	0.957	0.997	0.968
	Dubinin–Radushkevich	*q*_*m*_ (mg g^−1^)	5.537	11.678	7.547	24.863
		*K*_DR_ (mol^2^ J^−2^)	0.004	0.009	0.001	0.003
		*E* (kJ mol^−1^)	11.952	7.454	21.320	12.910
		*R*^2^	0.944	0.860	0.944	0.919
Nonlinear	Langmuir	*q*_m_ (mg g^−1^)	11.746	15.359	11.559	48.275
		*K*_L_ (L mg^−1^)	0.061	1.096	0.188	0.968
		*R*^2^	0.999	0.973	0.999	0.990
		*R*^2^_adj_	0.998	0.964	0.999	0.987
		RMSE	0.121	0.879	0.096	0.701
	Freundlich	1*/n*	0.649	0.387	0.478	0.187
		*K*_F_ (mg g^−1^) (L mg^−1^)^1/n^	0.967	7.698	2.522	12.450
		*R*^2^	0.997	0.993	0.988	0.999
		*R*^2^_adj_	0.995	0.990	0.984	0.999
		RMSE	0.080	0.389	0.227	0.229
	Temkin	*b*_T_ (J mol^−1^)	986.903	758.128	759.567	1033.791
		*A*_T_ (L g^−1^)	0.572	11.715	1.509	25.899
		*R*^2^	0.996	0.958	0.997	0.971
		*R*^2^_adj_	0.994	0.945	0.996	0.962
		RMSE	0.091	0.708	0.098	1.220
	Dubinin–Radushkevich	*q*_*m*_ (mg g^−1^)	5.751	12.102	7.761	24.636
		*K*_DR_ (mol^2^ J^−2^)	0.004	0.006	0.002	0.008
		*E* (kJ mol^−1^)	10.911	8.451	20.310	7.906
		*R*^2^	0.937	0.863	0.936	0.917
		*R*^2^_adj_	0.916	0.818	0.914	0.889
		RMSE	0.342	1.384	0.485	2.051

**Table 5:** The comparison of linear and nonlinear isotherm parameters for RB4 dye adsorptions on sawdust beads (SPB) and sawdust powder doped iron (III) oxide-hydroxide beads (SPFB).

Regression method	Isotherm model	Parameter	SPB	SPFB
Linear	Langmuir	*q*_m_ (mg g^−1^)	7.605	10.309
		*K*_L_ (L mg^−1^)	0.100	0.455
		*R*^2^	0.996	0.995
	Freundlich	1*/n*	0.635	0.394
		*K*_F_ (mg g^−1^) (L mg^−1^)^1/n^	0.895	1.418
		*R*^2^	0.988	0.998
	Temkin	*b*_T_ (J mol^−1^)	398.331	407.163
		*A*_T_ (L g^−1^)	0.299	0.730
		*R*^2^	0.954	0.985
	Dubinin–Radushkevich	*q*_*m*_ (mg g^−1^)	3.947	7.463
		*K*_DR_ (mol^2^ J^−2^)	0.005	0.001
		*E* (kJ mol^−1^)	9.901	20.412
		*R*^2^	0.945	0.967
Nonlinear	Langmuir	*q*_m_ (mg g^−1^)	7.536	10.161
		*K*_L_ (L mg^−1^)	0.102	0.470
		*R*^2^	0.996	0.987
		*R*^2^_adj_	0.995	0.982
		RMSE	0.060	0.232
	Freundlich	1*/n*	0.603	0.385
		*K*_F_ (mg g^−1^) (L mg^−1^)^1/n^	0.950	1.722
		*R*^2^	0.986	0.998
		*R*^2^_adj_	0.981	0.997
		RMSE	0.116	0.097
	Temkin	*b*_T_ (J mol^−1^)	396.218	410.654
		*A*_T_ (L g^−1^)	0.308	0.742
		*R*^2^	0.956	0.986
		*R*^2^_adj_	0.942	0.981
		RMSE	0.205	0.238
	Dubinin–Radushkevich	*q*_*m*_ (mg g^−1^)	4.108	7.712
		*K*_DR_ (mol^2^ J^−2^)	0.002	0.002
		*E* (kJ mol^−1^)	9.931	21.349
		*R*^2^	0.948	0.969
		*R*^2^_adj_	0.931	0.959
		RMSE	0.224	0.714

For lead adsorption, the adsorption patterns of SP and SPB corresponded to Langmuir isotherm with relating to a physical adsorption because their *R*^2^ values in both linear and nonlinear had higher than Freundlich, Temkin, and Dubinin–Radushkevich models. Therefore, Langmuir parameters of *q*_m_ and *K*_L_ values were used for explaining the adsorption pattern. Since *q*_m_ and *K*_L_ values of SPB were higher than SP, SPB had possibly higher lead removal efficiency with a high adsorption rate than SP correlated to the results of the batch experiment. For SPF and SPFB, their adsorption patterns corresponded to Freundlich isotherm with relating to a physiochemical adsorption because their *R*^2^ values in both linear and nonlinear had higher than Langmuir, Temkin, and Dubinin–Radushkevich models. Therefore, Freundlich parameters of *K*_F_ and 1/*n* values were used for explaining the adsorption pattern. *K*_F_ refers Freundlich adsorption constant which SPFB represented the highest *K*_F_ value, so SPFB had a higher adsorption rate than SPF. For a 1/*n* value, it is a constant depiction of the adsorption intensity which 0 < 1/*n* < 1 means the favorable adsorption isotherm, so both materials were favorable adsorption since their 1/*n* values in this range.

For RB4 dye adsorption, the adsorption pattern of SPB corresponded to Langmuir model relating to a physical adsorption whereas the adsorption pattern of SPFB corresponded to the Freundlich model correlated to a physicochemical adsorption from choosing the highest *R*^2^ value isotherm or closely to 1. These results corresponded to lead adsorption patterns that Langmuir and Freundlich isotherms were best-fitted models for explaining lead adsorption patterns of SPB and SPFB. Therefore, both lead and RB4 dye adsorption patterns of SPB and SPFB were physical and physicochemical adsorption processes, respectively.

Moreover, the results of both linear and nonlinear Langmuir, Freundlich, Temkin, and Dubinin–Radushkevich models of all sawdust materials were agreed with each other, so the plotting of both linear and nonlinear isotherm models were also recommended for correct data translations^42–44^.

Finally, the comparison of the maximum adsorption capacity (*q*_m_) value for lead and RB4 dye adsorptions by various adsorbents is illustrated in Table 6. For the comparison of lead removal, all sawdust materials in this study had higher *q*_m_ values than *q*_m_ values from *Picea smithiana* sawdust, sawdust-based cellulose nanocrystals, lemon peels, pomelo peels, and onion peels. In addition, SPFB had a higher *q*_m_ value than all studies in Table 6 except the studies of Hajam et al. (Dibetou sawdust activated by HNO_3_ and NaOH), Niu et al. (pine sawdust biochar modified with Mn-Zn ferrite), Aigbe and Kavaz (sawdust modified with zinc oxide), Liu et al. (sugarcane bagasse modified with Fe_3_O_4_), Zhao et al. (corncob biochar modified with CuFe_2_O_4_), Kang et al. (cassava stalks modified with Fe_3_O_4_), and Ahmadi et al. (melon peel). For the comparison of RB4 dye removal, both SPB and SPFB had higher *q*_m_ values than *q*_m_ values from bagasse, bagasse fly ash, and lemon peels demonstrated in Table 6. Therefore, all sawdust materials in this study were highly efficient materials for lead and RB4 dye adsorptions, and they are potential materials for application in industrial applications in the future, especially SPFB.

**Table 6:** Comparison of the maximum adsorption capacity for lead and RB4 dye adsorptions by various adsorbents.

Adsorbent materials	Dose	Contract time	Temperature (°C)	pH	Concentration (mg L^−1^)	*q*_m_ (mg g^−1^)	References
Lead adsorption							
*Picea smithiana* sawdust	20 g L^−1^	1 h	25	8	0–200	8.53	^45^
*Pinus halepensis* sawdust	10 g L^−1^	24 h	20–60	5–8	1–50	13.48	^46^
Dibetou sawdust	0.875 g	90 min		6	10–500	33.33	^37^
Dibetou sawdust activated by HNO_3_ and NaOH	0.875 g	47.5 min	25	6	10–500	61.73	^37^
Sawdust based cellulose nanocrystals	5 mg L^−1^		25–45	7	10–200	2.55	^47^
Sawdust biochar	0.1 g	24 h	25	6	10	17.57	^15^
Pine sawdust biochar modified with Mn–Zn ferrite	0.05 g	24 h	25	5	5–100	99.5	^48^
Sawdust modified with zinc oxide	0.05 g	100 min	25	8	100–600	92.59	^9^
Paddy husk biochar	0.1 g	24 h	25	6	10	14.20	^15^
Rice husk biochar	0.1 g			5–6	0–600	14.10–26.70	^49^
Coconut shells activated carbon doped Fe_2_O_3_	1.75 g L^−1^	24 h	22	6	5–100	11.90	^50^
Macadamia nutshells modified Cu–Mn	0.1 g	2 h	25–45	6	100	34.50	^51^
Pineapple stem	0.05 g	5 h	25	5	50–150	13.30	^52^
Pineapple stem modified oxalic acid	0.05 g	5 h	25	5	50–150	27.70	^52^
Plum fruit biomass	0.2 g	30 min	22	6	5–300	28.80	^53^
Apricot kernels fruit biomass	0.2 g	30 min	22	6	5–300	23.90	^53^
Bagasse	1 g	2 h	–	5.5	40–240	31.45	^17^
Sugarcane bagasse modified with Fe_3_O_4_	50 mg	4 h	25–55	7	10–500	116.7	^10^
Corncob biochar modified with CuFe_2_O_4_	20 mg	8 h	30–50	5	60–500	132.10–134.41	^54^
Cassava stalks modified with Fe_3_O_4_	2 g	1 h	45	6	50–400	163.93	^55^
Watermelon seed	0.5 g	6 h	28–60	2	20–200	18.55–19.42	^56^
Lemon peel powder	4 g	6 h	25	5	10–70	1.81	^18^
Lemon peel with iron (III) oxide-hydroxide powder	3 g	6 h	25	5	10–70	3.52	^8^
Lemon peel beads	5 g	6 h	25	5	10–70	3.16	^18^
Lemon peel with iron (III) oxide-hydroxide beads	2 g	5 h	25	5	10–70	5.67	^8^
Orange peels	0.5 g	2 h		5	10–100	40.05	^57^
Pomelo peels		3 h	30	2.5	10–30	2.14	^58^
Banana peels	0.5 g	2 h		5	10–100	37.69	^59^
Melon peels	1.5 g L^−1^	1 h	30	7	10–500	191.93	^19^
Onion peels	0.5 g	3 h	25	4	10–100	4.878	^60^
Onion peels modified with thioglycolic acid	0.5 g	3 h	25	4	10–100	6.173	^60^
SP	2 g	5 h	25	5	30–70	11.74	This study
SPF	1 g	3 h	25	5	30–70	15.92	This study
SPB	1.5 g	4 h	25	5	30–70	12.77	This study
SPFB	0.5 g	2 h	25	5	30–70	47.17	This study
RB4 dye adsorption							
Ayous wood sawdust	0.03 g	2 h	25		10–1400	415.10	^61^
Husk of agarwood fruit hydrogel beads	0.03 g		30		200–600	156.25–270.27	^62^
*Picea abies* Karst.bark with ZnCl_2_	1.5 g L^−1^		22	4	30–1000	59.00	^63^
*Picea abies* Karst.bark with KOH	1.5 g L^−1^		22	4	30–1000	339.15	^63^
Rice bran modified with SnO_2_/Fe_3_O_4_	1.5 g	3 h	60	3	10–200	200.40–218.82	^11^
Bagasse beads	2 g	12 h	70	3	30–90	3.17	^12^
Bagasse beads with mixed iron (III) oxide-hydroxide	3 g	9 h	70	3	30–90	3.77	^12^
Bagasse beads with mixed zinc oxide	3 g	12 h	60	3	30–90	3.18	^12^
Bagasse fly ash beads	3 g	12 h	70	3	30–90	5.57	^12^
Bagasse fly ash beads with mixed iron (III) oxide-hydroxide	2 g	3 h	50	3	30–90	10.28	^12^
Bagasse fly ash with beads mixed zinc oxide	2 g	12 h	40	3	30–90	6.78	^12^
Chicken eggshell beads	0.4 g	12 h	50	3	30–90	24.10	^14^
Chicken eggshell beads mixed iron (III) oxide-hydroxide	0.3 g	12 h	50	3	30–90	30.49	^14^
Chicken eggshell beads mixed zinc oxide	0.4 g	12 h	50	3	30–90	20.41	^14^
Duck eggshell beads	0.4 g	12 h	50	3	30–90	12.63	^14^
Duck eggshell beads mixed iron (III) oxide-hydroxide	0.3 g	12 h	50	3	30–90	25.97	^14^
Duck eggshell beads mixed zinc oxide	0.4 g	12 h	50	3	30–90	19.23	^14^
Lemon peel beads-doped iron (III) oxide-hydroxide	3 g	6 h	30	3	30–90	3.23	^13^
Lemon peel beads-doped zinc oxide	3 g	9 h	30	3	30–90	2.59	^13^
SPB	3 g	12 h	40	3	30–70	7.61	This study
SPFB	1.5 g	9 h	30	3	30–70	10.31	This study

### Adsorption kinetics for lead and RB4 dye adsorptions

**Figure 8:**
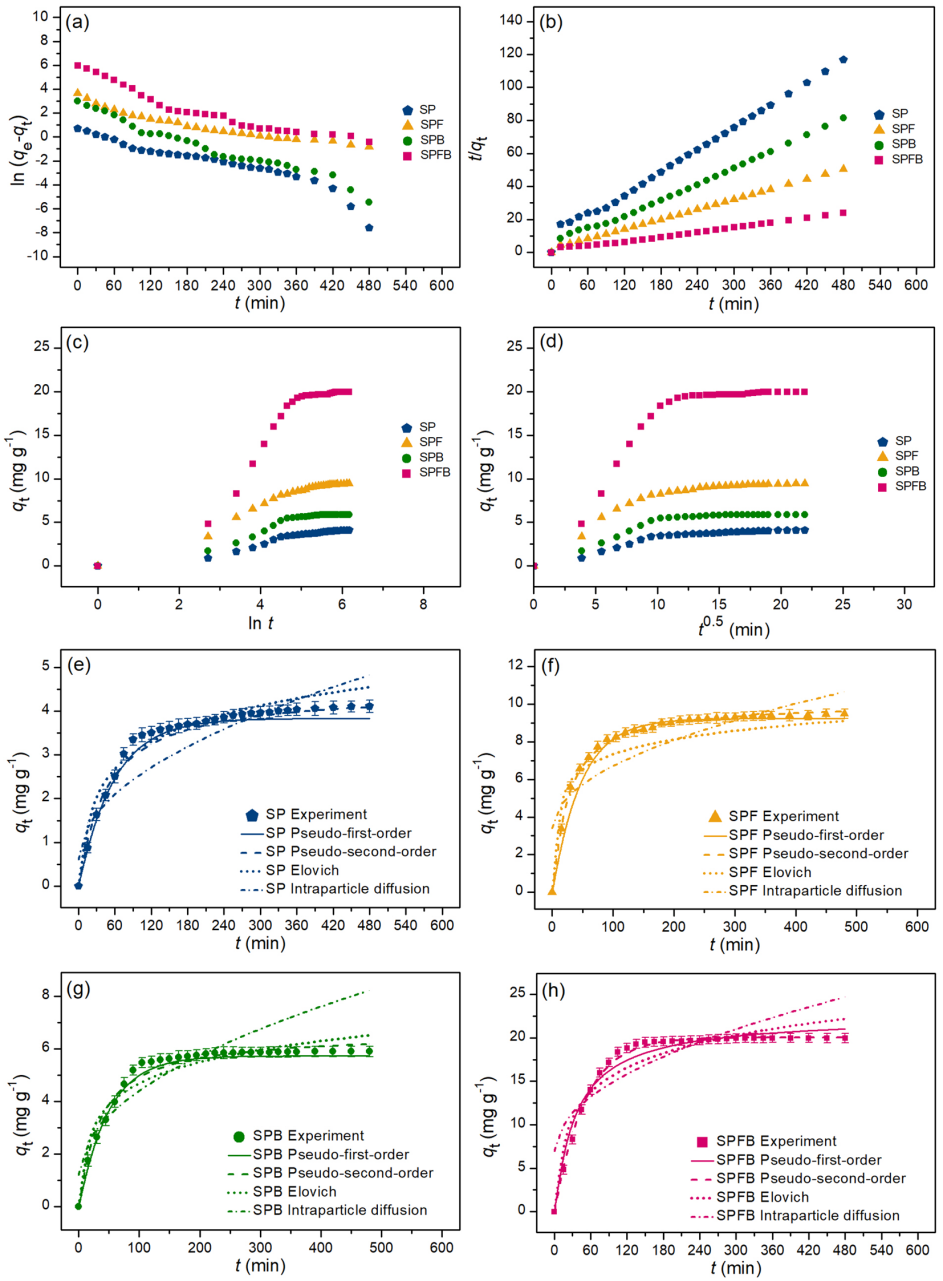
Graphs of (**a**) linear pseudo-first-order, (**b**) linear pseudo-second-order, (**c**) linear Elovich model (**d**) linear intraparticle diffusion, and (**e**–**h**) nonlinear kinetic models of sawdust powder (SP), sawdust powder doped iron (III) oxide-hydroxide (SPF), sawdust beads (SPB), and sawdust powder doped iron (III) oxide-hydroxide beads (SPFB) for lead adsorptions.

**Figure 9:**
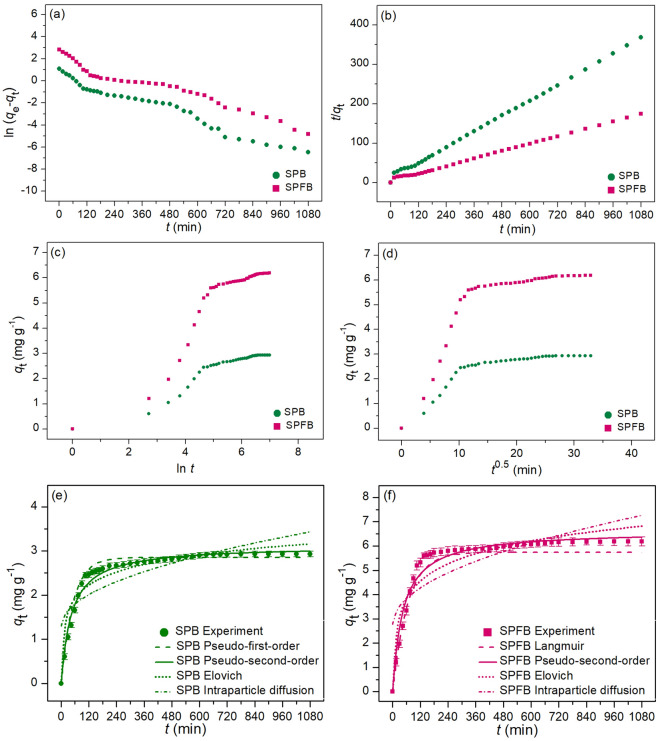
Graphs of (**a**) linear pseudo-first-order, (**b**) linear pseudo-second-order, (**c**) linear Elovich model (**d**) linear intraparticle diffusion, and (**e**,**f**) nonlinear kinetic models of sawdust beads (SPB) and sawdust powder doped iron (III) oxide-hydroxide beads (SPFB) for RB4 dye adsorptions.

The adsorption rates and mechanisms of lead adsorption by sawdust powder (SP), sawdust powder doped iron (III) oxide-hydroxide (SPF), sawdust beads (SPB), and sawdust powder doped iron (III) oxide-hydroxide beads (SPFB) and RB4 dye adsorption by sawdust beads (SPB) and sawdust powder doped iron (III) oxide-hydroxide beads (SPFB) were investigated through various the adsorption kinetics of pseudo-first-order kinetic model, pseudo-second-order kinetic model, Elovich model, and intraparticle diffusion in both linear and nonlinear models. For linear models, they were plotted by ln (*q*_e_ − *q*_t_) versus time (*t*), *t*/*q*_t_ versus time (*t*), *q*_t_ versus ln *t*, and *q*_t_ versus time (*t*^0.5^) for pseudo-first-order kinetic, pseudo-second-order kinetic, Elovich, and intraparticle diffusion models, respectively. For nonlinear models, they were plotted by *q*_t_ versus time (*t*). The plotting graphs of lead and RB4 dye adsorptions illustrated in Figs. 8a–h and 9a–f, respectively, and their adsorption kinetic parameters were demonstrated in Tables 7 and 8, respectively. Generally, the best-fit isotherm model for explaining the adsorption rate and mechanism of material is chosen from the high regression value (*R*^2^) which is close to 1^12^.

**Table 7:** The comparison of linear and nonlinear kinetic parameters for lead adsorptions on sawdust powder (SP), sawdust powder doped iron (III) oxide-hydroxide (SPF), sawdust beads (SPB), and sawdust powder doped iron (III) oxide-hydroxide beads (SPFB).

Regression method	Kinetic model	Parameter	SP	SPF	SPB	SPFB
Linear	Pseudo-first-order	*q*_e_ (mg g^−1^)	2.309	7.977	3.522	20.941
		*k*_1_ (min^−1^)	0.010	0.019	0.018	0.022
		*R*^2^	0.972	0.968	0.982	0.982
	Pseudo-second-order	*q*_e_ (mg g^−1^)	4.405	10.173	6.274	21.322
		*k*_2_ (g mg^−1^ min^−1^)	0.004	0.012	0.007	0.047
		*R*^2^	0.996	0.998	0.996	0.995
	Elovich	*α* (mg g^−1^ min^−1^)	5.477	6.465	5.944	7.327
		*β* (g mg^−1^)	1.646	0.440	0.863	0.267
		*R*^2^	0.936	0.848	0.848	0.901
	Intraparticle diffusion	*k*_i_ (mg g^−1^ min^−0.5^)	0.167	0.324	0.224	0.770
		*C*_i_ (mg g^−1^)	1.114	3.834	2.073	6.912
		*R*^2^	0.803	0.721	0.720	0.705
Nonlinear	Pseudo-first-order	*q*_e_ (mg g^−1^)	2.376	8.036	3.724	20.963
		*k*_1_ (min^−1^)	0.017	0.017	0.021	0.024
		*R*^2^	0.974	0.970	0.984	0.984
		*R*^2^_adj_	0.973	0.969	0.983	0.983
		RMSE	0.167	0.369	0.189	1.297
	Pseudo-second-order	*q*_e_ (mg g^−1^)	4.486	10.231	6.305	22.578
		*k*_2_ (g mg^−1^ min^−1^)	0.005	0.016	0.009	0.051
		*R*^2^	0.994	0.997	0.997	0.996
		*R*^2^_adj_	0.993	0.997	0.996	0.995
		RMSE	0.157	0.119	0.498	0.939
	Elovich	*α* (mg g^−1^ min^−1^)	5.632	6.540	6.053	7.493
		*β* (g mg^−1^)	1.784	0.453	0.889	0.272
		*R*^2^	0.939	0.851	0.851	0.906
		*R*^2^_adj_	0.937	0.845	0.846	0.902
		RMSE	0.256	0.823	0.821	1.572
	Intraparticle diffusion	*k*_i_ (mg g^−1^ min^−0.5^)	0.173	0.331	0.237	0.811
		*C*_i_ (mg g^−1^)	1.134	3.917	2.176	6.964
		*R*^2^	0.805	0.723	0.722	0.706
		*R*^2^adj	0.798	0.713	0.712	0.699
		RMSE	0.458	1.122	1.124	2.310

**Table 8:** The comparison of linear and nonlinear kinetic parameters for RB4 dye adsorptions on sawdust beads (SPB) and sawdust powder doped iron (III) oxide-hydroxide beads (SPFB).

Regression method	Kinetic model	Parameter	SPB	SPFB
Linear	Pseudo-first-order	*q*_e_ (mg g^−1^)	1.634	2.747
		*k*_1_ (min^−1^)	0.006	0.007
		*R*^2^	0.969	0.950
	Pseudo-second-order	*q*_e_ (mg g^−1^)	3.051	6.418
		*k*_2_ (g mg^−1^ min^−1^)	0.005	0.009
		*R*^2^	0.999	0.998
	Elovich	*α* (mg g^−1^ min^−1^)	0.783	0.872
		*β* (g mg^−1^)	2.128	1.223
		*R*^2^	0.903	0.869
	Intraparticle diffusion	*k*_i_ (mg g^−1^ min^−0.5^)	0.065	0.136
		*C*_i_ (mg g^−1^)	1.300	2.783
		*R*^2^	0.653	0.613
Nonlinear	Pseudo-first-order	*q*_e_ (mg g^−1^)	1.713	2.873
		*k*_1_ (min^−1^)	0.008	0.008
		*R*^2^	0.973	0.951
		*R*^2^_adj_	0.972	0.949
		RMSE	0.142	0.339
	Pseudo-second-order	*q*_e_ (mg g^−1^)	3.117	6.623
		*k*_2_ (g mg^−1^ min^−1^)	0.007	0.010
		*R*^2^	0.995	0.996
		*R*^2^_adj_	0.994	0.995
		RMSE	0.105	0.305
	Elovich	*α* (mg g^−1^ min^−1^)	0.859	0.894
		*β* (g mg^−1^)	2.213	1.234
		*R*^2^	0.904	0.871
		*R*^2^_adj_	0.901	0.867
		RMSE	0.229	0.550
	Intraparticle diffusion	*k*_i_ (mg g^−1^ min^−0.5^)	0.073	0.155
		*C*_i_ (mg g^−1^)	1.331	3.057
		*R*^2^	0.658	0.621
		*R*^2^adj	0.649	0.610
		RMSE	0.415	0.950

For lead adsorption, the adsorption rate and mechanism of sawdust materials corresponded to a pseudo-second-order kinetic model with relating to the chemisorption process because their *R*^2^ values in both linear and nonlinear pseudo-second-order kinetic models were higher than pseudo-first-order kinetic, Elovich, and intraparticle diffusion models. Therefore, the adsorption kinetic parameters of *q*_e_ and *k*_2_ were used for explaining the adsorption rate and mechanism. Their adsorption capacities (*q*_e_) of a pseudo-second-order kinetic model were arranged in order from high to low of SPFB > SPF > SPB > SP correlated to the results of batch experiments and adsorption isotherm. For a *k*_2_ value, it is the pseudo-second-order kinetic rate constant in which SPFB demonstrated the highest value than other adsorbents. As a result, SPFB had higher lead adsorption with a fast reaction than other materials.

For RB4 dye adsorption, the adsorption rate and mechanism of SPB and SPFB corresponded to a pseudo-second-order kinetic model similar to lead adsorption. In addition, *q*_e_ and *k*_2_ values of SPFB had also higher than SPB, so SPFB had higher RB4 dye adsorption than SPB corresponding to lead adsorption of SPB and SPFB. Therefore, both lead and RB4 dye adsorption rates and mechanisms of SPB and SPFB were explained by a physicochemical adsorption process.

Finally, the results of both linear and nonlinear pseudo-first-order, pseudo-second-order kinetic, Elovich, and intraparticle diffusion models of all sawdust materials were consistent with each other, so the plotting graphs of both linear and nonlinear kinetic models were also recommended for protecting mistake data translations^42–44^.

### Desorption experiments for lead and RB4 dye adsorptions

The desorption experiments were used to investigate the feasibility of the reuse of sawdust powder (SP), sawdust powder doped iron (III) oxide-hydroxide (SPF), sawdust beads (SPB), and sawdust powder doped iron (III) oxide-hydroxide beads (SPFB) because this is a necessary point to estimate the cost and economic feasibility of industrial applications.

**Figure 10:**
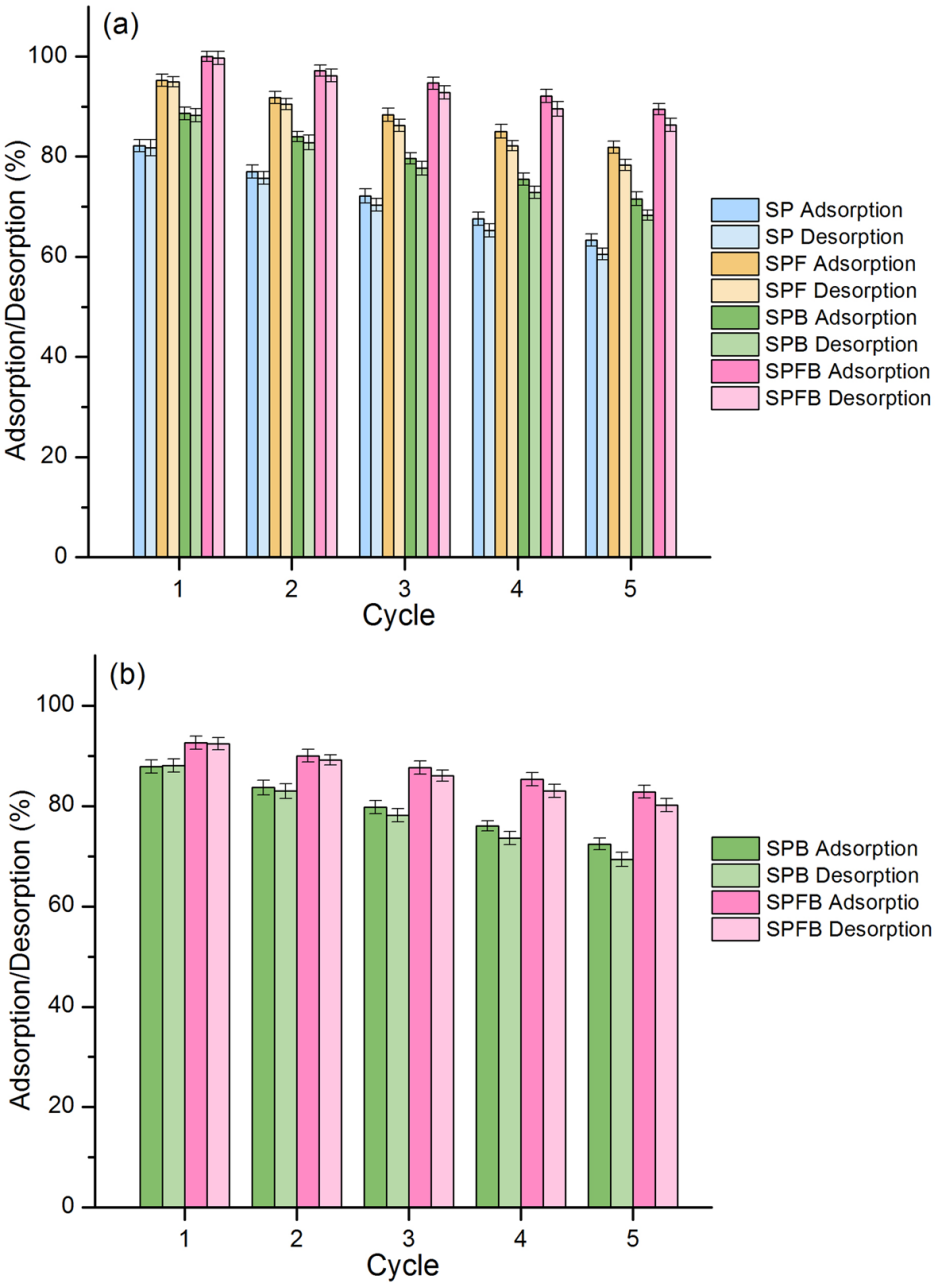
The desorption experiments of (**a**) sawdust powder (SP), sawdust powder doped iron (III) oxide-hydroxide (SPF), sawdust beads (SPB), and sawdust powder doped iron (III) oxide-hydroxide beads (SPFB) for lead removal and (**b**) sawdust beads (SPB) and sawdust powder doped iron (III) oxide-hydroxide beads (SPFB) for RB4 dye removal.

For Lead adsorption, SP, SPF, SPB, and SPFB for 5 cycles of adsorption–desorption were applied to confirm their abilities, and their results are illustrated in Fig. 10a. For SP, it could be reused in 5 cycles with high adsorption and desorption in ranges of 63.33–82.16% and 60.54–81.76%, respectively which adsorption and desorption were decreased by approximately 19% and 21%, respectively. For SPF, it also confirmed to be reusability in 5 cycles with high adsorption and desorption in ranges of 81.83–95.23% and 78.30–94.93%, respectively which adsorption and desorption were decreased by approximately 13% and 17%, respectively. For SPB, it could be reused in 5 cycles with high adsorption and desorption in ranges of 71.58–88.62% and 68.33–88.27%, respectively which adsorption and desorption were decreased by approximately 17% and 20%, respectively. For SPFB, it also confirmed to be reusability in 5 cycles with high adsorption and desorption in ranges of 89.45–100% and 86.33–99.75%, respectively which adsorption and desorption were decreased by approximately 11% and 13%, respectively. Therefore, sawdust materials are potential materials for lead adsorption with the reusability of more than 5 cycles by more than 63%, and they can be further applied to industrial applications.

For RB4 dye adsorption, SPB and SPFB for 5 cycles of adsorption–desorption were applied to confirm their abilities, and their results are illustrated in Fig. 10b. For SPB, it could be reused in 5 cycles with high adsorption and desorption in ranges of 72.45–87.84% and 69.36–88.09%, respectively which adsorption and desorption were decreased by approximately 15% and 19%, respectively. For SPFB, it also confirmed to be reusability in 5 cycles with high adsorption and desorption in ranges of 82.85–92.63% and 80.15–92.43%, respectively which adsorption and desorption were decreased by approximately 10% and 12%, respectively. Therefore, SPB and SPFB are potential materials for RB4 dye removal with the reusability of more than 5 cycles by more than 72%, and they can be applied to industrial applications in the future.

## The possible mechanisms of lead and dye adsorptions by sawdust materials

**Figure 11:**
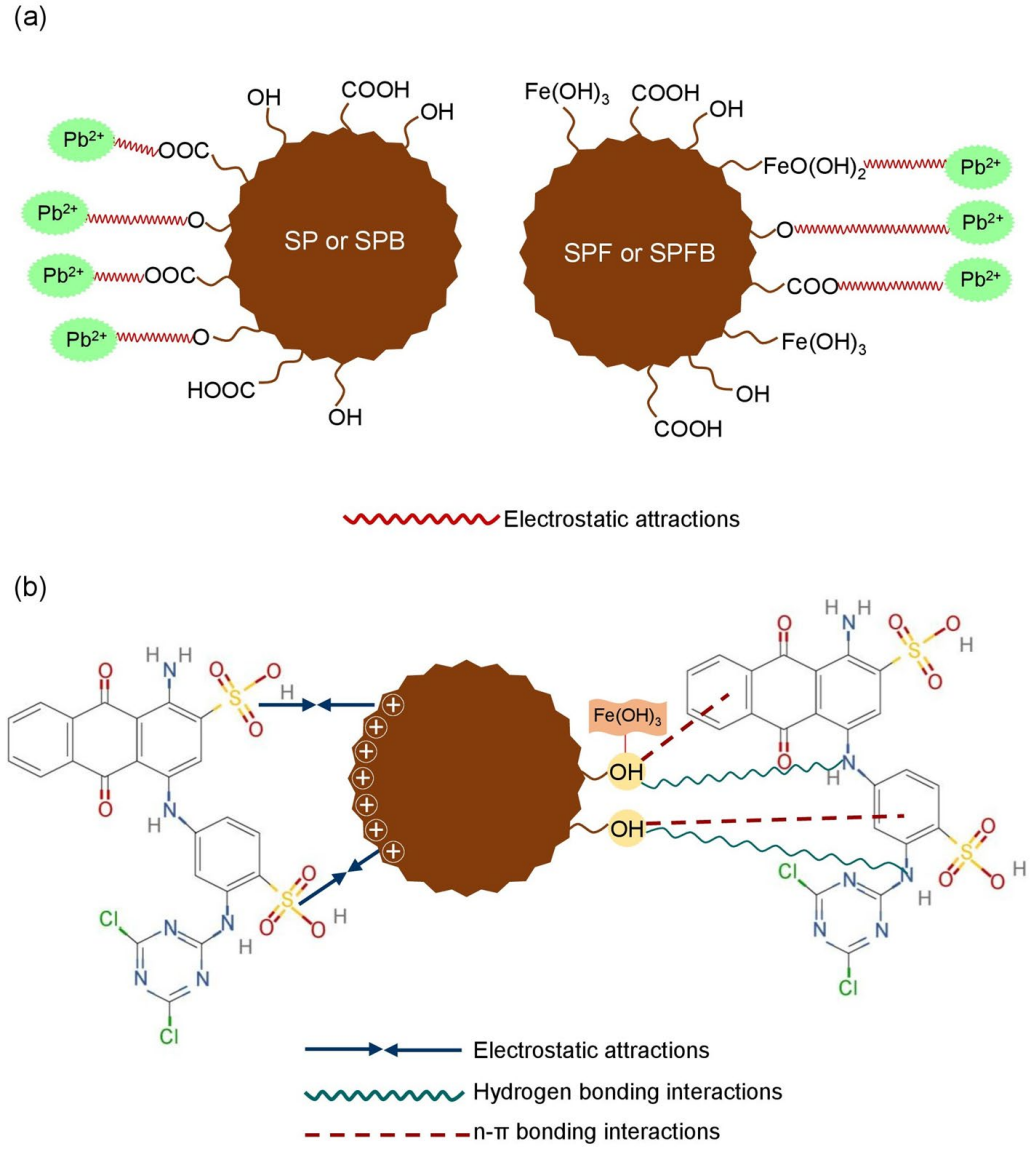
Possible mechanisms of (**a**) sawdust powder (SP), sawdust powder doped iron (III) oxide-hydroxide (SPF), sawdust beads (SPB), and sawdust powder doped iron (III) oxide-hydroxide beads (SPFB) for lead adsorption and (**b**) sawdust beads (SPB) and sawdust powder doped iron (III) oxide-hydroxide beads (SPFB) for RB4 dye adsorption.

The possible mechanisms of lead and RB4 dye adsorptions on sawdust materials are demonstrated in Fig. 11a,b. For lead adsorption, the cellulose, hemicellulose, pectin, lignin, a hydroxyl group (–OH), methyl groups (C–H), aromatic ring represented lignin (C=C), and alcohol and carboxylic acid of lignin and hemicellulose (C–H) were the main structure and chemical functions groups of sawdust materials. The carboxyl group (–COOH) was also demonstrated in sawdust beads (SPB and SPFB) by forming the complex compound between SP or SPF with sodium alginate. In addition, the complex compound between the surface of SPF or SPFB and iron (III) oxide-hydroxide was formed to be SP∙Fe(OH)_3_ or SPB∙Fe(OH)_3_ by a process of electron sharing with hydroxyl groups of sawdust. Therefore, the possible mechanism of lead adsorption by sawdust materials might occur from donating a proton (H^+^) from carboxyl groups (–COOH) or hydroxyl groups (–OH) or SP∙Fe(OH)_3_ or SPB∙Fe(OH)_3_ of main chemical compounds or complex compounds to be –COO or –O or FeO(OH)_2_ for capturing lead (II) ions (Pb^2+^) by instead of H^+^ from a process of electrostatic interactions^64^ shown in Fig. 11a.

For RB4 dye adsorption, the main structure and chemical groups of SPB and SPFB were the same as mentioned above, but the possible mechanism of RB4 dye adsorption used a different explanation based on Ngamsurach et al.^12^ shown in Fig. 11b. Three possible mechanisms of electrostatic attractions, hydrogen bonding interactions, and n–π bonding interactions were used for explaining RB4 dye adsorptions by SPB and SPFB. For electrostatic interactions, the surface of SPB or SPFB adsorbed RB4 dye molecule from the interaction between the positively charged hydroxy group (–OH) of their surface and the negatively charged sulfonate groups (–SO_3_^−^) of RB4 dye molecules. For hydrogen bonding interactions, the nitrogen (N) in the RB4 dye structure was caught by hydrogen ions (H^+^) in the hydroxyl group (–OH) of SPB or SPFB^14^. Finally, the n–π bonding interactions occurred by the interaction of oxygen bond (–O) in the hydroxyl group (–OH) in SPB or SPFB and the aromatic rings in RB4 dye molecules^6^.

## Conclusion

Four sawdust materials of sawdust powder (SP), sawdust powder doped iron (III) oxide-hydroxide (SPF), sawdust beads (SPB), and sawdust powder doped iron (III) oxide-hydroxide beads (SPFB) were successfully synthesized for lead or RB4 dye removals in an aqueous solution. SPFB demonstrated higher specific surface area (11.020 m^2^ g^−1^) and smaller pore size (3.937 nm) than other materials, and the results illustrated that the addition of iron (III) oxide-hydroxide into sawdust materials increased the specific surface area and pore volume while the pore diameter size was decreased. The surface morphologies of SP and SPF were irregular shapes with heterogeneous fiber structures whereas SPB and SPFB had spherical shapes with coarse surfaces. Carbon (C) and oxygen (O) were found in all materials whereas iron (Fe) was only found in the materials with the addition of iron (III) oxide-hydroxide (SPF and SPFB). Four main function groups of O–H, C–H, C=C, and C–O were detected in all materials. For batch experiments, the optimum conditions of SP, SPF, SPB, and SPFB for lead adsorptions were 2 g, 5 h, pH 5, 50 mg L^−1^, 1 g, 3 h, pH 5, 50 mg L^−1^, 1.5 g, 4 h, pH 5, 50 mg L^−1^, and 0.5 g, 2 h, pH 5, 50 mg L^−1^, respectively, and the optimum conditions of SPB and SPFB for RB4 dye adsorptions were 3 g, 12 h, 40 °C, pH 3, 50 mg L^−1^ and 1.5 g, 9 h, 30 °C, pH 3, 50 mg L^−1^, respectively. Since SPFB demonstrated the highest lead or RB4 dye removal than other materials, adding iron (III) oxide-hydroxide and changing material form helped to improve material efficiencies for lead or RB4 dye adsorptions. For adsorption isotherms, SP and SPB corresponded to Langmuir model correlated to physical adsorption whereas SPF and SPFB corresponded to the Freundlich model relating to a physicochemical adsorption process. For the kinetic study, all materials corresponded to a pseudo-second-order kinetic model related to a chemisorption process with heterogenous adsorption. For desorption experiments, all materials could be reused more than 5 cycles with high lead removal of 63%, and SPB and SPFB also could be reused more than 5 cycles with high RB4 dye removal of 72%. Therefore, all sawdust materials were high potential materials for lead or dye adsorption in an aqueous solution, and SPFB demonstrated the highest lead and RB4 dye removals. Therefore, SPFB was suitable for wastewater treatment in industrial applications.

For future works, the real wastewater with contaminated lead or RB4 dye should be investigated to confirm the ability of sawdust materials, and the continuous flow study also needs to study for further industrial applications.

## Materials and methods

### Raw material

Sawdust (*Pterocarpus indicus*) was obtained from a local sawmill in Khon Kaen province, Thailand.

### Chemicals

All chemicals were analytical grades (AR) without purification before use. For material synthesis, ferric chloride hexahydrate (FeCl_3_·6H_2_O) (LOBA, India), sodium hydroxide (NaOH) (RCI Labscan, Thailand), sodium alginate (NaC_6_H_7_O_6_) (Merck, Germany), and calcium chloride dehydrate (CaCl_2_·2H_2_O) (RCI Labscan, Thailand) were used. For preparing wastewater samples, lead nitrate (Pb(NO_3_)_2_) (QRëC, New Zealand), and reactive blue 4 (RB4) dye (Sigma-Aldrich, Germany) were used. The chemical characteristic and structure of RB4 dye demonstrated in Table 9. For pH adjustment, 1% NaOH and 1% HNO_3_ (Merck, Germany) were used.

**Table 9:** The chemical characteristic and structure of RB4 dye.

Properties and characteristics of dye	
Chemical structure	
Chemical name	Reactive blue 4 (RB4)
IUPAC name	1-amino-4-[3-[(4,6-dichloro-1,3,5-triazin-2-yl)amino]-4-sulfoanilino]-9,10-dioxoanthracene-2-sulfonic acid
Chemical formula	C_23_H_14_Cl_2_N_6_O_8_S_2_
Chemical class	Anionic dye
Molecular weight (g/mol)	637.43
(*λ*_max_) (nm)	595
Color index number	61205

### Synthesis of four sawdust materials

**Figure 12:**
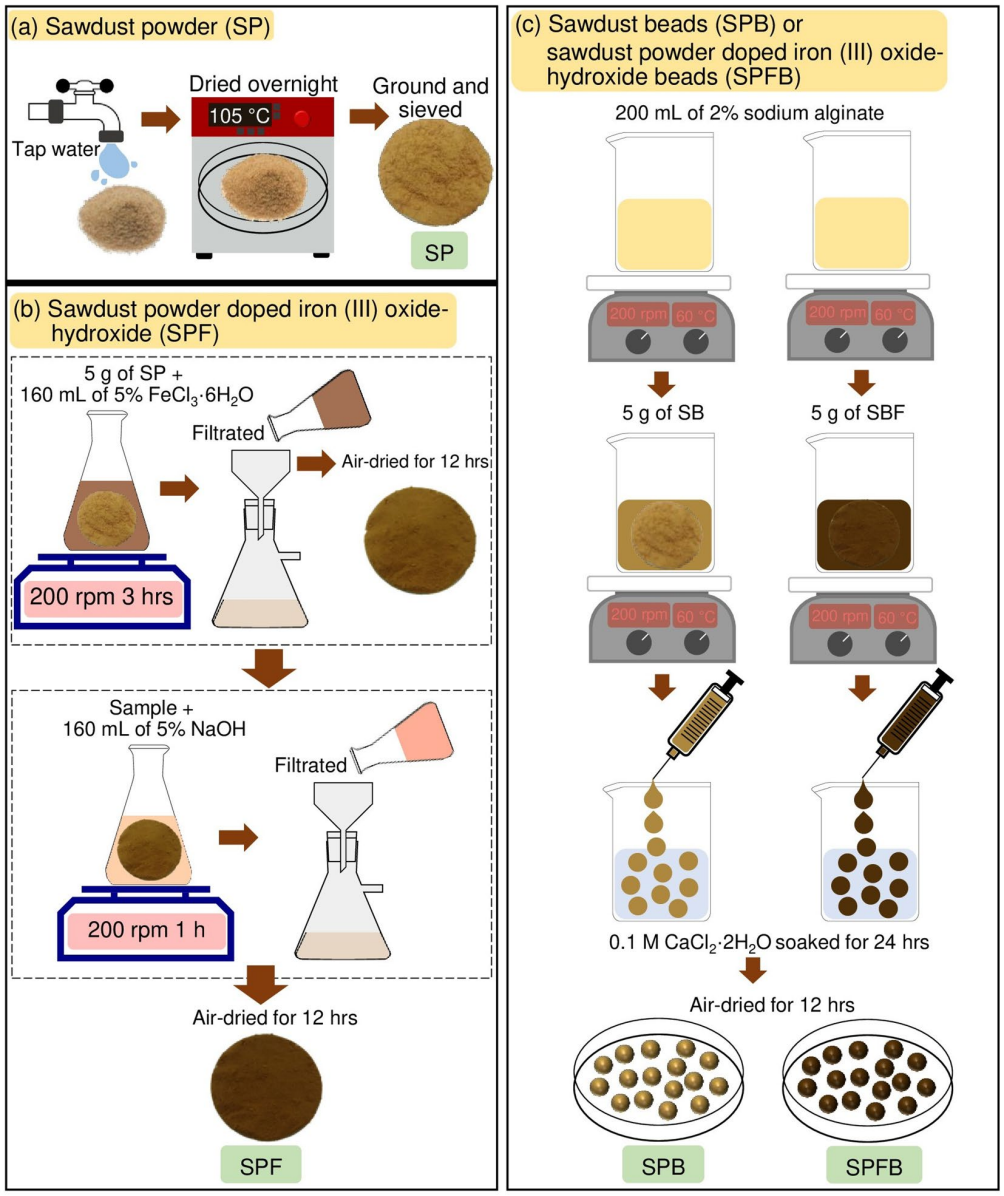
Flow diagrams of synthesis methods of (**a**) sawdust powder (SP), (**b**) sawdust powder doped iron (III) oxide-hydroxide (SPF), and (**c**) sawdust beads (SPB) and sawdust powder doped iron (III) oxide-hydroxide beads (SPFB).

The flow diagrams of synthesis methods of four sawdust materials which were sawdust powder (SP), sawdust powder doped iron (III) oxide-hydroxide (SPF), sawdust beads (SPB), and sawdust powder doped iron (III) oxide-hydroxide beads (SPFB) based on Threepanich and Praipipat^8^ are demonstrated in Fig. 12a–c and the details were clearly explained below.

#### The synthesis of sawdust powder (SP)

Firstly, sawdust was washed with tap water to remove contaminants, and then it was dried overnight in a hot air oven (Binder, FED 53, Germany) at 105 °C. Then, it was ground and sieved to a size of 125 µm. Finally, it was kept in a desiccator before use called sawdust powder (SP).

#### The synthesis of sawdust powder doped iron (III) oxide-hydroxide (SPF)

Firstly, 5 g of SP were added to 500 mL of Erlenmeyer flask containing 160 mL of 5% FeCl_3_·6H_2_O, and they were mixed by an orbital shaker (GFL, 3020, Germany) of 200 rpm for 3 h. Secondly, they were filtrated and air-dried at room temperature for 12 h. Then, they were added to 500 mL of Erlenmeyer flask containing 160 mL of 5% NaOH, and they were mixed by an orbital shaker of 200 rpm for 1 h. After that, they were filtered and air-dried at room temperature for 12 h. Finally, they were kept in a desiccator before use called sawdust powder doped iron (III) oxide-hydroxide (SPF).

#### The synthesis of sawdust beads (SPB) or sawdust powder doped iron (III) oxide-hydroxide beads (SPFB)

Firstly, 5 g of SP or SPF were added to 500 mL of a beaker containing 200 mL of 2% sodium alginate, and then they were homogeneously mixed and heated by a hot plate (Ingenieurbüro CAT, M. Zipperer GmbH, M 6, Germany) at 60 °C with a constant stirring of 200 rpm. Secondly, they were dropped by drop by using a 10 mL syringe with a needle size of 1.2 × 40 mm into 250 mL of 0.1 M CaCl_2_·2H_2_O. The beaded samples were soaked in 0.1 M CaCl_2_·2H_2_O for 24 h, and then they were filtered and rinsed with DI water. After that, they were air-dried at room temperature for 12 h and kept in a desiccator before use called sawdust beads (SPB) or sawdust powder doped iron (III) oxide-hydroxide beads (SPFB).

### Characterizations of sawdust materials

Various characterized techniques of Brunauer–Emmett–Teller (BET) (Bel, Bel Sorp mini X, Japan) by isothermal nitrogen gas (N_2_) adsorption–desorption at 77.3 K and degas temperature of 80 °C for 6 h, field emission scanning electron microscopy and focus ion beam (FESEM-FIB) with energy dispersive X-ray spectrometer (EDX) (FEI, Helios NanoLab G3 CX, USA), and Fourier transform infrared spectroscopy (FT-IR) (Bruker, TENSOR27, Hong Kong) were used for investigating the specific surface area, pore volume, pore size, surface morphology, chemical compositions, and chemical functional groups of sawdust powder (SP), sawdust powder doped iron (III) oxide-hydroxide (SPF), sawdust beads (SPB), and sawdust powder doped iron (III) oxide-hydroxide beads (SPFB).

### Batch adsorption experiments

#### Batch experiments for lead removal

A series of batch adsorption experiments were designed to investigate the effect of dose, contact time, pH, and concentration on lead removal efficiency by sawdust powder (SP), sawdust powder doped iron (III) oxide-hydroxide (SPF), sawdust beads (SPB), and sawdust powder doped iron (III) oxide-hydroxide beads (SPFB). The differences in dose from 0.5 to 3 g, contact time from 1 to 6 h, pH values of 1, 3, 5, 7, 9, 11, and lead concentration from 30 to 70 mg L^−1^ with the control condition of initial lead concentration of 50 mg L^−1^, a sample volume of 200 mL, a shaking speed of 200 rpm, a temperature of 25 °C were applied. The lowest value of each affecting factor with the highest lead removal efficiency was selected as the optimum value, and that value was applied to the next affecting factor study. Lead concentrations were analyzed by an atomic adsorption spectrophotometer (PerkinElmer, PinAAcle 900 F, USA), and triplicate experiments were conducted to confirm the results. Lead removal in the percentage (%) to calculate the following Eq. (1).

1 $${\text{Lead removal efficiency}}\;(\% ) = (C_{0} - C_{{\text{e}}} )/C_{0} \times \, 100$$

where *C*_0_ is the initial lead concentration (mg L^−1^), and *C*_e_ is the final lead concentration (mg L^−1^).

#### Batch experiments for RB4 dye removal

A series of batch adsorption experiments were designed to investigate the effect of dose, contact time, temperature, pH, and concentration on RB4 dye removal efficiency by sawdust beads (SPB) and sawdust powder doped iron (III) oxide-hydroxide beads (SPFB). The differences in dose from 0.5 to 3 g, contact time of 3, 6, 9, 12, 15, 18 h, temperature from 30 to 80 °C, pH values of 1, 3, 5, 7, 9, 11, and RB4 dye concentration from 30 to 70 mg L^−1^ with the control condition of initial RB4 dye concentration of 50 mg L^−1^, a sample volume of 200 mL, a shaking speed of 150 rpm, and a contact time of 12 h were applied. The lowest value of each affecting factor with the highest RB4 dye removal efficiency was selected as the optimum value, and that value was applied to the next affecting factor study. Dye concentrations were analyzed by UV–Vis spectrophotometer (Hitachi, UH5300, Japan) at a maximum wavelength of 595 nm, and triplicate experiments were conducted to confirm the results. Dye removal in the percentage (%) to calculate the following Eq. (2).

2 $${\text{Dye removal efficiency}}\;(\% ) = (C_{0} - C_{{\text{e}}} )/C_{0} \times 100$$

where *C*_0_ is the initial dye concentration (mg L^−1^), and *C*_e_ is the final dye concentration (mg L^−1^).

### Adsorption isotherms

The adsorption patterns of sawdust powder (SP), sawdust powder doped iron (III) oxide-hydroxide (SPF), sawdust beads (SPB), and sawdust powder doped iron (III) oxide-hydroxide beads (SPFB) are investigated by adsorption isotherms for explaining that are the adsorption process of monolayer or multi-layer or heat or thermodynamic. Linear and nonlinear Langmuir, Freundlich, Temkin, and Dubinin–Radushkevich models are used to analyze followed Eqs. (3)–(10)^65–68^.

Langmuir isotherm:

3 $${\text{Linear:}}\quad C_{{\text{e}}} /q_{{\text{e}}} = 1/q_{{\text{m}}} K_{{\text{L}}} + C_{{\text{e}}} /q_{{\text{m}}}$$

4 $${\text{Nonlinear:}}\quad q_{{\text{e}}} = q_{{\text{m}}} K_{{\text{L}}} C_{{\text{e}}} /1 + K_{{\text{L}}} C_{{\text{e}}}$$

Freundlich isotherm:

5 $${\text{Linear:}}\quad \log q_{{\text{e}}} = \log K_{{\text{F}}} + 1/n\log C_{{\text{e}}}$$

6 $${\text{Nonlinear:}}\quad q_{{\text{e}}} = K_{{\text{F}}} C_{{\text{e}}}^{1/n}$$

Temkin isotherm:

7 $${\text{Linear:}}\quad q_{{\text{e}}} = RT/b_{{\text{T}}} \;\ln\; A_{{\text{T}}} + RT/b_{{\text{T}}}\; \ln\; C_{{\text{e}}}$$

8 $${\text{Nonlinear:}}\quad q_{{\text{e}}} = RT/b_{{\text{T}}}\; \ln\; A_{{\text{T}}} C_{{\text{e}}}$$

Dubinin–Radushkevich isotherm:

9 $${\text{Linear:}}\quad \ln \;q_{{\text{e}}} = \, \ln \;q_{{\text{m}}} {-}K_{{{\text{DR}}}} \varepsilon^{2}$$

10 $${\text{Nonlinear:}}\quad q_{{\text{e}}} = q_{{\text{m}}} \exp ( - K_{{{\text{DR}}}} \varepsilon^{2} )$$

where *C*_e_ is the equilibrium of lead or dye concentration (mg L^−1^), *q*_e_ is the amount of adsorbed lead or dye on sawdust materials (mg g^−1^), *q*_m_ is indicated as the maximum amount of lead or dye adsorption on adsorbent materials (mg g^−1^), *K*_L_ is the adsorption constant (L mg^−1^). *K*_F_ is the constant of adsorption capacity (mg g^−1^) (L mg^−1^)^1/n^, and 1/*n* is the constant depicting the adsorption intensity. *R* is the universal gas constant (8.314 J mol^−1^ K^−1^), *T* is the absolute temperature (K), *b*_T_ is the constant related to the heat of adsorption (J mol^−1^), and *A*_T_ is the equilibrium binding constant corresponding to the maximum binding energy (L g^−1^). *q*_m_ is the theoretical saturation adsorption capacity (mg g^−1^), *K*_DR_ is the activity coefficient related to mean adsorption energy (mol^2^ J^−2^), and *ε* is the Polanyi potential (J mol^−1^). Graphs of linear Langmuir, Freundlich, Temkin, and Dubinin–Radushkevich isotherms were plotted by *C*_e_*/q*_e_ versus *C*_e,_ log *q*_e_ versus log *C*_e_, *q*_e_ versus ln *C*_e_, and ln *q*_e_ versus *ε*^2^, respectively whereas graphs of their nonlinear were plotted by *q*_e_ versus *C*_e_*.*

For adsorption isotherm experiments, 2 g of SP or 1 g of SPF, or 1.5 g of SPB or 0.5 g of SPFB was added to 200 mL Erlenmeyer flasks with variable lead concentrations from 30 to 70 mg L^−1^ with the control condition of sample volume of 200 mL, a shaking speed of 200 rpm, pH 6, a temperature of 25 °C, and a contact time of 5 h for SP, 3 h for SPF, 4 h for SPB, and 2 h for SPFB for studying lead adsorption. For studying RB4 dye adsorption, 3 g of SPB or 1.5 g of SPFB was added to 200 mL Erlenmeyer flasks with variable RB4 dye concentrations from 30 to 70 mg L^−1^ with the control condition of a sample volume of 200 mL, a shaking speed of 150 rpm, pH 7, a temperature of 40 °C for SPB and 30 °C SPFB, and a contact time of 12 h.

### Adsorption kinetics

The adsorption mechanisms of sawdust powder (SP), sawdust powder doped iron (III) oxide-hydroxide (SPF), sawdust beads (SPB), and sawdust powder doped iron (III) oxide-hydroxide beads (SPFB) are determined by various adsorption kinetics which were linear and nonlinear pseudo-first-order kinetic, pseudo-second-order kinetic, Elovich, and intraparticle diffusion models calculated by Eqs. (11)–(17)^69–72^.

Pseudo-first-order kinetic model:

11 $${\text{Linear:}}\quad \ln \;(q_{{\text{e}}} - q_{{\text{t}}} ) = \ln\; q_{{\text{e}}} {-} \, k_{1} t$$

12 $${\text{Nonlinear:}}\quad q_{{\text{t}}} = q_{{\text{e}}} (1 - e^{{ - k_{1} t}} )$$

Pseudo-second-order kinetic model:

13 $${\text{Linear:}}\quad t/q_{{\text{t}}} = 1/k_{2} q_{{\text{e}}}^{2} + \, \left( {t/q_{{\text{e}}} } \right)$$

14 $${\text{Nonlinear:}}\quad q_{{\text{t}}} = k_{2} q_{{\text{e}}}^{2} t/\left( {1 + \, q_{{\text{e}}} k_{2} t} \right)$$

Elovich model:

15 $${\text{Linear:}}\quad q_{{\text{t}}} = \, 1/\beta \;\ln \;\alpha \beta + \, 1/\beta \;\ln \;t$$

16 $${\text{Nonlinear:}}\quad q_{{\text{t}}} = \beta \;\ln \;t + \beta \;\ln \;\alpha$$

Intraparticle diffusion model:

17 $${\text{Linear and nonlinear:}}\quad q_{{\text{t}}} = k_{{\text{i}}} t^{0.5} + C_{{\text{i}}}$$

where *q*_e_ is the amount of adsorbed lead or dye on adsorbent materials (mg g^−1^), *q*_t_ is the amount of adsorbed lead or dye at the time (*t*) (mg g^−1^), *k*_1_ is a pseudo-first-order rate constant (min^−1^), and *k*_2_ is a pseudo-second-order rate constant (g mg^−1^ min^−1^)^73^. *α* is the initial adsorption rate (mg g^−1^ min^−1^) and *β* is the extent of surface coverage (g mg^−1^). *k*_i_ is the intraparticle diffusion rate constant (mg g^−1^ min^−0.5^) and *C*_i_ is the constant that gives an idea about the thickness of the boundary layer (mg g^−1^). Graphs of linear pseudo-first-order, pseudo-second-order, Elovich, and intraparticle diffusion models were plotted by ln (*q*_e_ − *q*_t_) versus time (*t*), *t*/*q*_t_ versus time (*t*), *q*_t_ versus ln *t*, and *q*_t_ versus time (*t*^0.5^), respectively whereas their nonlinear graphs were plotted by the capacity of lead or dye adsorbed by sawdust materials at the time (*q*_t_) versus time (*t*).

For adsorption kinetic experiments, 10 g of SP or 5 g of SPF, or 7.5 g of SPB or 2.5 g of SPFB was added to 1000 mL of breaker with the control condition of the initial lead concentration of 50 mg L^−1^, a sample volume of 1000 mL, a shaking speed of 200 rpm, pH 5, a temperature of 25 °C, and a contact time of 8 h for studying lead adsorption. For studying RB4 dye adsorption, 15 g of SPB or 7.5 g of SPFB was added to 1000 mL of breaker with the control condition of initial RB4 dye concentration of 50 mg L^−1^, a shaking speed of 150 rpm, pH 3, a temperature of 40 °C for SPB and 30 °C SPFB, and a contact time of 18 h.

### Desorption experiments

The possible material reusability is an important factor for considering adsorbents of industrial applications, so the desorption experiments are designed to examine by studying five adsorption–desorption cycles to confirm the abilities of sawdust powder (SP), sawdust powder doped iron (III) oxide-hydroxide (SPF), sawdust beads (SPB), and sawdust powder doped iron (III) oxide-hydroxide beads (SPFB) for lead adsorption or sawdust beads (SPB) and sawdust powder doped iron (III) oxide-hydroxide beads (SPFB) for RB4 dye adsorption. For lead adsorption, the saturated sawdust materials were added to 500 mL of Erlenmeyer flask containing 200 mL of 0.5 M HNO_3_ solution, then it was shaken by an incubator shaker (New Brunswick, Innova 42, USA) at 200 rpm for 6 h. Then, they were washed with deionization water and dried at room temperature, and sawdust materials are ready for the next adsorption cycle. For RB4 dye adsorption, the saturated sawdust materials were added to 500 mL of Erlenmeyer flask containing 200 mL of 0.01 M NaOH solution, then it was shaken by an incubator shaker at 150 rpm for 15 h with a temperature of 30 °C. Then, they were washed with deionization water and dried at room temperature, and sawdust materials are ready for the next adsorption cycle. The desorption efficiency in percentage is calculated following Eq. (18).

18 $${\text{Desorption}}\;(\% ) = (q_{{\text{d}}} {/}q_{{\text{a}}} ) \times 100$$

where *q*_d_ is the amount of lead or dye desorbed (mg mL^−1^) and *q*_a_ is the amount of lead or dye adsorbed (mg mL^−1^).

## Data Availability

The datasets used and/or analyzed during the current study are available from the corresponding author upon reasonable request.
